# Spider phylosymbiosis: divergence of widow spider species and their tissues’ microbiomes

**DOI:** 10.1186/s12862-020-01664-x

**Published:** 2020-08-18

**Authors:** Sara J. Dunaj, Brian R. Bettencourt, Jessica E. Garb, Robert M. Brucker

**Affiliations:** 1 Department of Biological Sciences, University of Massachusetts Lowell, Lowell, MA USA; 2 Triplet Therapeutics, Cambridge, MA USA; 3 The Rowland Institute of Harvard University, Cambridge, MA USA

**Keywords:** Phylosymbiosis, Host-microbe interactions, Black widow spiders, Common house spider, Hologenome, Metatranscriptomics, Arthropod evolution, Microbiome

## Abstract

**Background:**

Microbiomes can have profound impacts on host biology and evolution, but to date, remain vastly understudied in spiders despite their unique and diverse predatory adaptations. This study evaluates closely related species of spiders and their host-microbe relationships in the context of phylosymbiosis, an eco-evolutionary pattern where the microbial community profile parallels the phylogeny of closely related host species. Using 16S rRNA gene amplicon sequencing, we characterized the microbiomes of five species with known phylogenetic relationships from the family Theridiidae, including multiple closely related widow spiders (*L. hesperus, L. mactans, L. geometricus, S. grossa,* and *P. tepidariorum*).

**Results:**

We compared whole animal and tissue-specific microbiomes (cephalothorax, fat bodies, venom glands, silk glands, and ovary) in the five species to better understand the relationship between spiders and their microbial symbionts. This showed a strong congruence of the microbiome beta-diversity of the whole spiders, cephalothorax, venom glands, and silk glands when compared to their host phylogeny. Our results support phylosymbiosis in these species and across their specialized tissues. The ovary tissue microbial dendrograms also parallel the widow phylogeny, suggesting vertical transfer of species-specific bacterial symbionts. By cross-validating with RNA sequencing data obtained from the venom glands, silk glands and ovaries of *L. hesperus, L. geometricus, S. grossa,* and *P. tepidariorum* we confirmed that several microbial symbionts of interest are viably active in the host*.*

**Conclusion:**

Together these results provide evidence that supports the importance of host-microbe interactions and the significant role microbial communities may play in the evolution and adaptation of their hosts.

## Background

Microbial communities play diverse and important roles in host organism biology, including influencing host nutrition and metabolism [1], immune function [2], animal behavior [1–3], and speciation [2–4]. Recent advances in molecular biology techniques and Next Generation Sequencing (NGS) technologies have allowed for sequencing microbiome samples at much lower costs and higher depth than in the past [5]. Consequently, the study of the “Hologenome” or a host’s genome plus their microbiome as a codependent unit is expanding to include important work across multiple disciplines (Human Microbiome Project, host-microbe interactions and toxicity, arthropod evolution and adaptation) [2, 6].

Arthropod microbiome research also includes evidence supporting arthropod speciation by symbiosis and the importance of studying the host genome and its microbiota in its entirety [3, 6]. For instance, host-microbe interactions influence host development and cause hybrid lethality in Jewel wasps (*Nasonia*) [4, 7]. Further, microbial communities within and across three closely related and environmentally controlled *Nasonia* species exhibit a phylogenetically distinct pattern that mirrors their hosts’ phylogeny [4]. This evolutionary host-microbe relationship provides strong evidence, along with other similar studies, to support a recently proposed hypothesis known as phylosymbiosis [7, 8]. Phylosymbiosis describes the ecological and evolutionary relationship of the microbiomes across related host species, where the microbial community profile parallels the phylogeny of closely related host species and maintains an “ancestral signal” [7, 8]. Recent research suggests that phylosymbiosis is common and can also play a role in host fitness and health. For example, deer mice inoculated with microbial communities from more distantly related species had lower food digestibility and jewel wasps that received transplants of interspecific microbiota had reduced survival compared to those exposed to their own intraspecific microbiota [8].

Microbiome research on non-insect arthropods, such as spiders, is limited and has mainly focused on PCR-based sequencing assays targeting specific, well characterized arthropod symbionts known to have an impact on arthropod fitness, reproductive behavior and isolation such as *Wobachia*, *Rickettsia*, *Spiroplasma*, and *Cardinium*, [9–19]. Comprehensive investigation of spider microbiomes has only been conducted in a few studies [12, 20], but the distinct evolutionary pattern of phylosymbiosis and existence of evolutionarily significant spider-microbe relationships have not been evaluated. Moreover, the degree to which spider species and different tissues harbor unique microbial communities is poorly known.

Black widow spiders (genus *Latrodectus*) provide a particularly suitable system to understand how microbes influence spider evolution. The availability of a well-resolved *Latrodectus* phylogeny facilitates tests of phylosymbiosis [21]. Additionally, this clade of spiders is medically significant because their venom contains neurotoxic latrotoxin proteins, making black widow spider venom hazardous to humans [21–23]. While latrotoxins are not known from spiders outside of the black widow family Theridiidae [23], recently Bordenstein and Bordenstein sequenced phage WO, that commonly infects *Wolbachia,* and concluded this viral genome encoded a latrotoxin C-terminal domain horizontally transferred from the black widow spider [24]. This study also confirmed the presence of *Wolbachia* in *Latrodectus geometricus* using targeted PCR. Similarly, Goodacre et al. [17] used targeted PCR to show an unidentified *Latrodectus* species was infected with *Wolbachia* and *Rickettsia,* but not *Spiroplasma*. This provides limited but intriguing evidence to suggest the presence of a diverse microbial community within *Latrodectus* species and the potential for genetic exchange with their microbial symbionts.

The purpose of this study is to characterize the microbiomes of widow spiders using high-throughput metagenomic sequencing and to evaluate if there is evidence of phylosymbiosis across closely related species in the genus *Latrodectus,* its sister genus *Steatoda* and the more distantly related common house spider (*Parasteatoda tepidariorum*). We hypothesize that the microbial communities of these spider species will diverge in a pattern mirroring their phylogeny and that each specialized tissue type will contain its own unique complement of microbial community members. Specifically, the microbial communities will not be randomly assembled but instead will be host-specific and will match the host phylogenetic pattern. Additionally, we expect that metabolically active and functional microbial symbionts would also be detectable via RNA sequencing of these spider species’ specialized tissues. Therefore, analysis of publicly available RNA sequencing data for the venom, silk, and ovary glands from *L. hesperus*, *L. geometricus*, *S. grossa*, and *P. tepidariorum* can provide support and validation for the functionally viable microbial community members of interest observed within the 16S sequencing data. Together these comparative analyses provide important insights into the evolution of spider-microbe relationships and the hologenome of these medically significant spiders.

## Results

### Diversity, host, and tissue specificity of spider microbiota

**Fig. 1:**
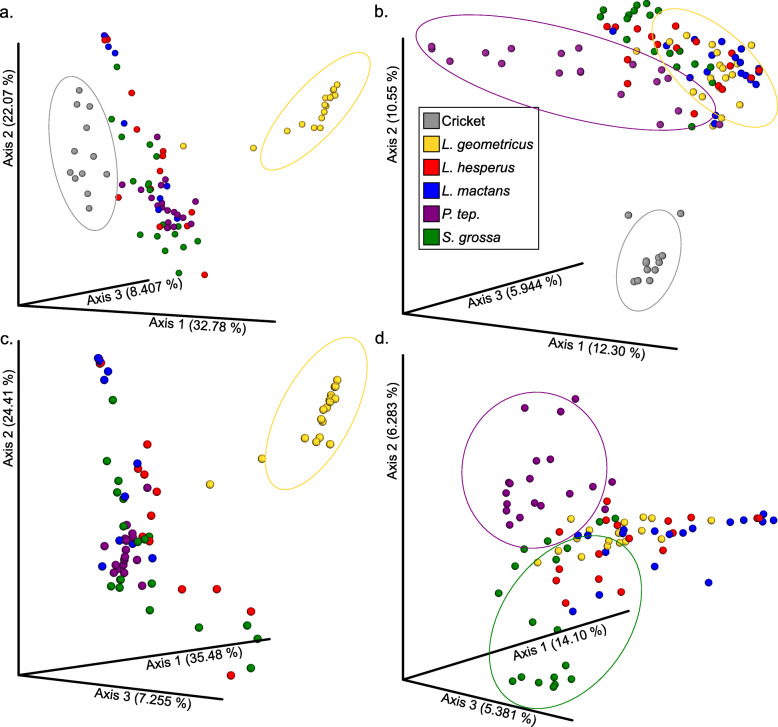
Beta-diversity principal coordinate analysis plots for whole animal samples. Weighted UniFrac plot with cricket samples (**a**) *- L. geometricus* samples cluster together within blue oval and crickets in red oval. Un-weighted UniFrac plot with cricket samples (**b**) - *P. tepidariorum* samples cluster together within purple oval, over half the *S. grossa* samples cluster together within yellow oval, and crickets cluster separately in red oval. Weighted UniFrac plot without cricket samples (**c**) - *L. geometricus* samples cluster together within red oval. Un-weighted UniFrac plot without cricket samples (**d**) -*P. tepidariorum* samples cluster together within green oval, over half the *S. grossa* samples cluster together within purple oval

The microbiome of each spider species included in this study (5 species in family Theridiidae) was evaluated from whole spider samples (3 per each species, 15 whole samples) and at a tissue-specific level (3 animal/ tissue sets per species, 5 tissues per set – cephalothorax, venom glands, ovary, silk glands, and fat tissue, 75 tissue samples). Crickets (12 whole), the prey items/ food source used for these spiders, were also included in this study to determine if their microbial communities affect the microbial composition and diversity of the host spider species. In total, including controls, 109 samples were processed through the 16S amplicon sequencing and analysis workflow (Fig. S1). The results from the alpha diversity tests (Fig. S2A & C, Faith’s Phylogenetic Diversity and Shannon Index respectively) showed that we achieved sufficient depth of sequencing coverage (≥5000 reads per sample) to measure OTU (Operational Taxonomic Unit) diversity, as the diversity per spider sample plateaued by 7000 sequences. Additionally, even after filtering sequences for quality and contamination, all but one of our spider samples had greater than 5000 reads per sample. We utilized these results to select the depth of coverage passed into the core phylogenetic diversity plug-in command in QIIME to generate EMPeror PCoA clustering plots for each UniFrac (weighted and unweighted) and Bray-Curtis dissimilarity distance matrices [25]. In each of the beta-diversity clustering PCoA plots, the spiders’ prey items (crickets) clearly cluster together and separately from all spider samples (Fig. 1a & b). The *L. geometricus* samples (whole and each tissue type replicate) segregate from other samples in the weighted UniFrac PCoA plots (Fig. 1a & c), while *P. tepidariorum* and many of *S. grossa* samples tend to cluster separately in the un-weighted UniFrac plots (Fig. 1b & d). In other words, the relative abundance (weighted UniFrac) of the dominant microbial community members in *L. geometricus* affects how the whole spider and tissues samples cluster together for this species. In contrast, the microbial community members in *S. grossa* and *P. tepidariorum* samples segregate when the abundances are not taken into account (presence – absence only) when generating UniFrac distance matrixes for PCoA plotting. Also, the *P. tepidariorum* whole spider samples clustered closely with other *P. tepidariorum* whole spider samples from different MiSeq runs. A small number (*n* = 7) of our negative controls had a low number of reads; of those that had some OTUs assigned, the number of reads (35–1035 reads, and one PCR control at 5689 reads), all were well below the number of reads retained after rarefaction (7032 cutoff) for diversity analyses run across all sample types and thus removed from downstream analyses.

**Fig. 2:**
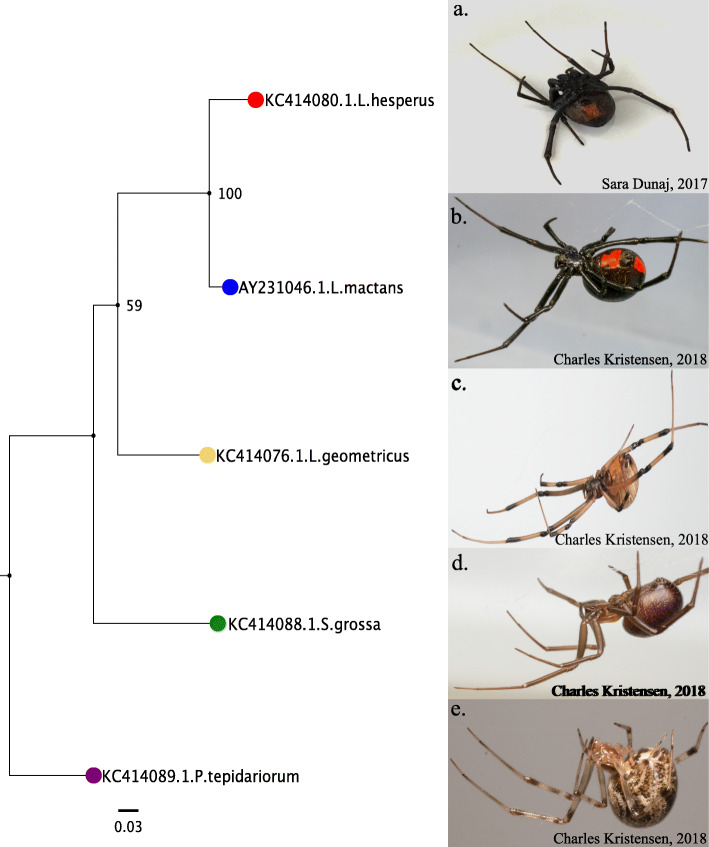
Taxa Plots Per Tissue Type and Species. Major microbial constituents (> 2%) within each whole spider and tissue sample for each species (replicates grouped via mean-ceiling). Sample types as follows: Whole Spiders, Cephalothorax, Venom Glands, Ovary, Silk Glands, and Fat Tissue. Widow anatomy illustration by S. Dunaj, 2018

The resulting assigned taxonomies for each group of spider samples were visualized with R to evaluate the composition and relative abundances of the more dominant (> 2% relative abundance) microbial community members within each tissue per species. The resulting tissue microbial community profiles vary in the level of diversity between spider species, where *P. tepidariorum* and *S. grossa* samples tended to have greater overall microbial community diversity compared to the *Latrodectus* species samples (Fig. 2 & Fig. S3). This variance of diversity is also observed in each of the alpha-diversity boxplots (Fig. S4A - D), where *P. tepidariorum* and *S. grossa* samples tend to have higher diversity indexes for their microbial communities as compared to *Latrodectus* species samples.

All of the *L. geometricus* samples were dominated by *Candidatus Rhabdochlamydia* (genus), by relative abundance*.* According to the SILVA database, this 16S sequence is specific to brown widows. This microbial symbiont had a broad range in abundance within the *L. geometricus* samples; at the lowest, average, relative abundance, 61% of the microbial community in fat tissues and up to 95% of whole spider samples (91% of ovary, 83% of silk glands, 76% of venom glands, 73% of cephalothorax) at the highest relative abundance. *Gilliamella* is a major bacterial symbiont that is found across many of the spider samples*.* This symbiont primarily dominates *L. mactans* and *L. hesperus* samples. The relative abundances of *Gilliamella* in *L. mactans* samples are as follows: 91% in ovary, 79% in whole spiders, 68% in silk glands, 62% in venom glands, 54% in fat tissue, and 35% in cephalothorax. *Gilliamella* also is a major microbial community member constituent within *L. hesperus* samples and is observed at the following relative abundances: 68% in silk glands, 40% in venom glands, 40% in cephalothorax, 30% in ovary, 27% in whole spiders, and 25% in fat tissue. *Gilliamella* is also observed to be a dominant (27%) bacterial community member in *S. grossa* fat tissue. Another prominent bacterial symbiont, *Spiroplasma,* is found to be a dominant community member within *L. hesperus* whole spiders, with an average relative abundance of 40%. *Wolbachia* is also observed in all the spider species and tissue types, except for in *P. tepidariorum*. The average relative abundance of *Wolbachia* ranges from low (< 1%) in some species to a high of 8% in *L. hesperus* ovary tissue. *Wolbachia* is also observed as a rare (< 1%) microbial community member within ovary samples of *L. geometricus*, *L. mactans* and *S. grossa*. *Wolbachia* is also detected as a significant symbiont in other tissue samples at the following relative abundances: 2.5% in whole *L. mactans,* 3% in *L. hesperus* silk glands, 3% in *S. grossa* silk glands and cephalothorax, and 2% in *L. geometricus* venom glands (0.8% in *L. hesperus* and 0.3% in *S. grossa* venom glands as well). Other major microbial community members that are found across several spider species and their tissues include *Bartonella*, *Ralstonia*, *Acinetobacter*, *Delftia*, *Rhizobiales*, *Sphingomonas*, *Propionibacterium*, and *Sphingomonadaceae.*

To determine the bacterial taxa unique to specialized organs, we analyzed the replicate grouped (mean-ceiling) master sample taxa tables from QIIME2–2018.4 (Level 6 – Genera) with a custom python script that computes the unique OTUs for each tissue type. Results show that there are two detectable microbial community members unique to venom glands and shared across all spider species: *Psychrobacter* and *Variovorax* (Table S4A lists all venom specific microbiota). There are also bacterial taxa unique to silk glands (Table S4B). One of these, *Candidatus Peribacteria,* is detected in multiple silk glands from two different spider species (*S. grossa* and *P. tepidariorum*). Several bacterial symbionts were observed to be unique to ovaries, including *Candidatus Falkowbacteria,* which was detected in both *L. geometricus* and *L. mactans* ovaries (see Table S5A for full listing of ovary microbiota). Lastly, Table S5B lists the microbiota specific to fat tissue, where *Gemmatimonas* was detected in both *P. tepidariorum* and *L. geometricus* fat tissue samples.

### RNA sequencing versus 16S amplicon sequencing

Our analysis of public RNA sequencing data from silk, venom, and ovary glands across multiple widow-related species found 30 bacterial symbionts/ community members were concordant with our generated 16S amplicon sequencing dataset (Tables 1 and 2). Overall, there are 94 shared OTUs observed between the 16S and RNA sequencing datasets and a large number of bacterial reads (taxonomic hits in CosmosID) within the RNA sequencing data for each sample type, ranging from 10,512 to 1,123,096 (Table 1). All of the bacterial hits/ OTUs from the RNA sequencing data that did not match our 16S dataset are listed in Table S6. Several of the top microbial constituents that did match between these datasets include the following: *Achromobacter, Acinetobacter, Bradyrhizobium, Burkholderiales, Methylobacterium, Propionibacteriaceae, Pseudomonas, Ralstonia, Sphingobium, Sphingomonas, Stenotrophomonas,* and *Wolbachia* (Table 2). *Pseudomonas, Ralstonia* and *Staphylococcus* are detected in the RNA-seq data across all four spider species. The following microbial community members are found in both the silk and venom glands of *P. tepidariorum*: *Achromobacter, Acinetobacter, Propionibacteriaceae,* and *Stenotrophomonas.* Also*, S. grossa* has twenty-one microbial community members found across both datasets and two of which are observed across the silk, venom, and ovary glands - *Methylobacterium* and *Staphylcoccus*. The venom glands of *L. geometricus* has three bacterial constituents found across both sets of sequencing data: *Sphingomonas*, *Streptococcus*, and *Veillonella. L. hesperus* has both *Escherichia* and *Sphingobium* within its venom glands. Within the 16S and RNA sequencing data for *L. hesperus* and *L. geometricus,* the venom glands for both species contain *Wolbachia, Streptococcus, Staphylococcus* and *Pseudomonas*.

**Table 1:** Summary of OTUs observed in RNA sequencing verses 16S amplicon sequencing of Silk, Venom, and Ovary glands in Widow related spiders

Sample ID	Tissue Type	16 s OTUs	RNAseq OTUs	Bacterial Hits (RNAseq)	Shared OTUs
*L. hesperus*	Silk Glands	55	1	10,512	0
*L. hesperus*	Venom Glands	79	24	152,364	12
*L. hesperus*	Ovary	68	20	39,829	6
*L. geometricus*	Silk Glands	53	8	47,646	4
*L. geometricus*	Venom Glands	73	18	1,181,879	10
*L. geometricus*	Ovary	71	10	519,983	6
*S. grosa*	Silk Glands	159	8	1,123,096	6
*S. grosa*	Venom Glands	180	26	1,122,123	16
*S. grosa*	Ovary	142	32	354,499	16
*P. tepidariorum*	Silk Glands	98	12	191,609	5
*P. tepidariorum*	Venom Glands	130	34	112,093	13
*P. tepidariorum*	Ovary	96	1	44,772	0

**Table 2:**
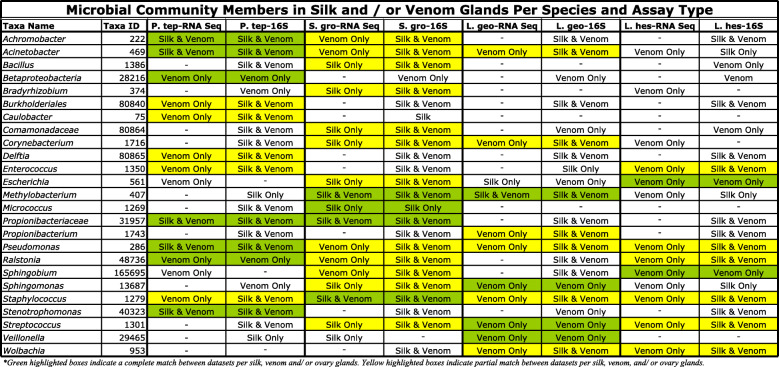
RNA sequencing verses 16S amplicon sequencing of silk, venom, and ovary glands from widow-related spiders

### Phylosymbiotic microbial community assembly

**Fig. 3:**
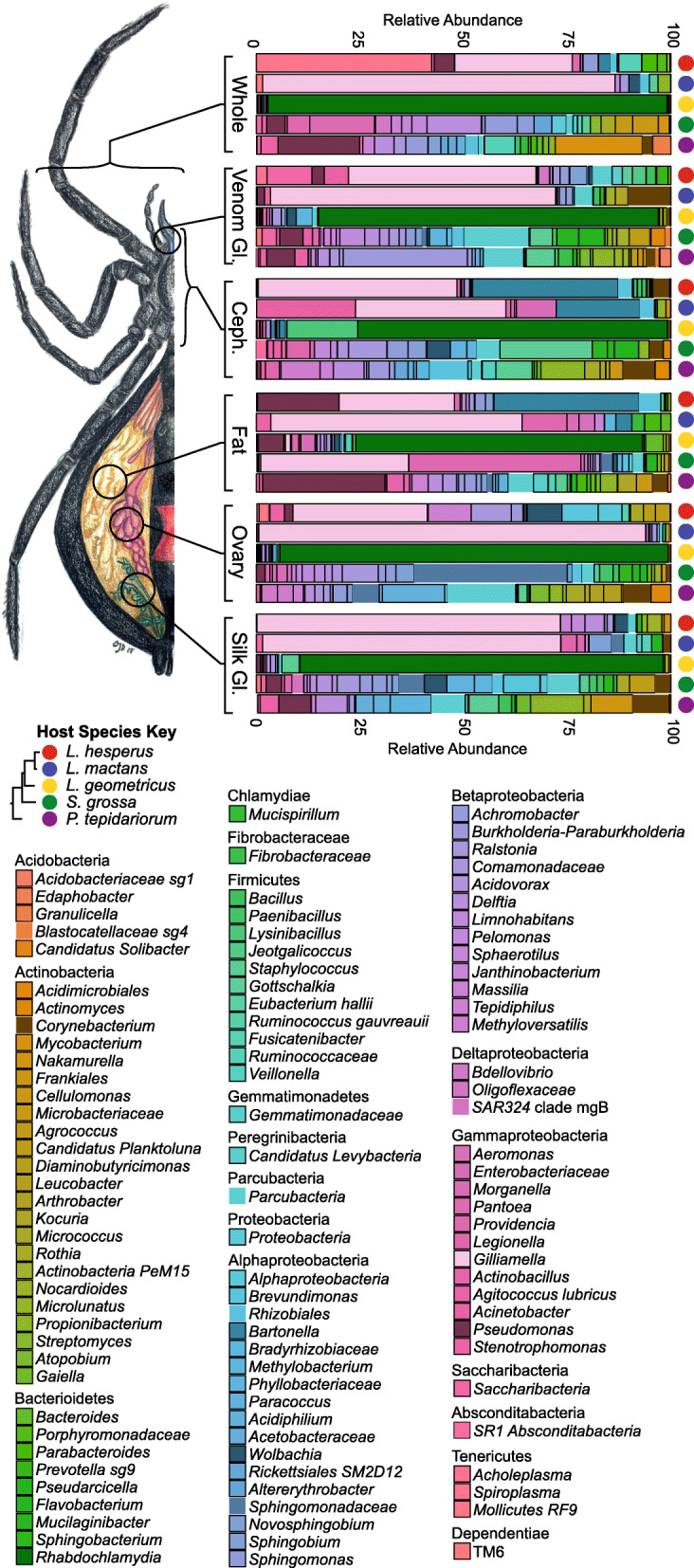
Widow Related Spider Phylogeny. Rooted (*P. tepidariorum*)*,* bootstrapped (10,000 iterations) phylogenetic tree from selected spider mtCOI genes (accession numbers in each branch label). Substitution model – GTR + γ. **a** Photograph of *L. hesperus* (S. Dunaj, 2017). Photographs of *L. mactans* (**b**), *L. geometricus* (**c**), *S. grossa* (**d**) and *P. tepidariorum* (**e**) (C. Kristensen, 2018)

In order to test for phylosymbiosis across our spider species and their tissues, a host phylogeny with at least four species of interest is required. A maximum likelihood phylogenetic tree (Fig. 3) was generated with RAxML based on the mitochondrial gene COI [26] utilizing the highest ranking Akaike Information Criterion (AIC) substitution model – GTR + γ (also the highest ranking models based on the Bayesian Information Criterion - BIC). This model is also within the AIC and BIC 100% confidence intervals determined by JModel Test 2 [27]. The resulting spider species phylogenetic tree was rooted with the *P. tepidariorum* branch, based on earlier phylogenies [23] (Fig. 3).

**Fig. 4:**
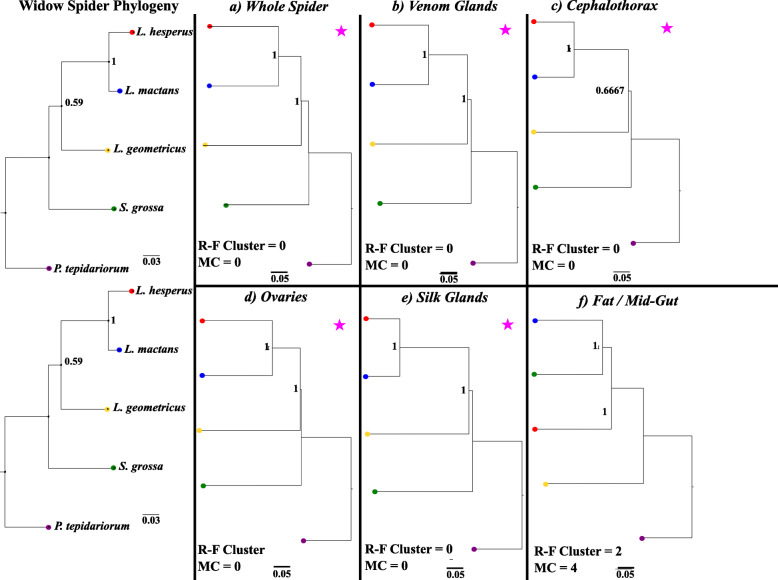
Phylosymbiosis between Widow Phylogeny and Bray-Curtis Microbiota Dendrogram Relationships. Congruency between widow related spider phylogeny and beta-diversity dendrograms was measured via Robinson-Foulds Cluster (R-F Cluster) metric, where a value of 0 = complete congruence and 2 = incomplete incongruence. Matching Cluster (MC) Metric also accounts for incongruency between closely related branches of rooted trees and a value of 0 also indicates complete congruence between compared trees. Pink asterisks are utilized to denote phylosymbiotic relationships between the widow phylogeny and microbial dendrogram

We then compared the distances between this reference widow phylogenetic tree to each resulting beta-diversity dendrogram generated from every set of grouped tissue and whole spider samples with TreeCmp [28]. Congruency between the host phylogeny and each beta-diversity dendrogram was evaluated by the resulting Robinson-Foulds Cluster and Matching Cluster measurements (Fig. 4 and Fig. S4), where the resulting scale for these metrics ranged from a value of 0 to 4 (with 0 equating to complete congruence) [8, 28]. Congruency arises from the corresponding/mirrored relationship between the branches of the host phylogenetic tree and the microbial beta-diversity tree. These beta-diversity dendrograms are generated from the clustering of the distance matrix values derived from each respective beta-diversity test. This congruency is indicative of a phylosymbiotic microbial community assembly across related species / host clade [3, 8]. We observe evidence for phylosymbiosis (host-microbiota congruency) across the following spider sample sets with the Bray-Curtis microbial beta-diversity dendrograms (Fig. 4): whole spiders, cephalothorax, venom glands, silk glands and ovaries. Host-microbiota congruency is also observed for several of these tissue sets and whole spiders in the weighted UniFrac and Jaccard beta-diversity dendrograms to host phylogeny comparisons, which can be viewed in Fig. S4. Interestingly, phylosymbiosis is not observed across the fat/ mid-gut tissues, the ovary tissues in the weighted UniFrac dendrogram or in any of the unweighted UniFrac dendrograms. An Adonis PERMANOVA test indicates significant impacts of species and tissue on beta diversity (F = 8.761037291 and 1.25112113, *P* = 0.000999 and 0.04495505, respectively), but no significant interaction (model: diversity = species * tissue).

## Discussion

In this study, we uncovered several key insights into widow spider microbial communities and provided support for phylosymbiotic patterns of these communities across several members of the Theridiidae family/widow spider host clade. We observed species-specific clustering of spider microbiome samples. Specifically, we detected a significant clustering pattern with the Weighted-UniFrac beta-diversity test for *L. geometricus* samples, where the majority of these samples were segregated from other spider species samples (Fig. 1a-c). We also found that *P. tepidariorum* and *S. grossa* have a greater diversity of microbial community members across whole spider and tissue specific samples, as compared to the *Latrodectus* species included in this study (Fig. 2). Additionally, OTUs corresponding to known arthropod symbionts were found across both 16S and RNA-seq datasets, validating the presence of active, functional microbial symbionts within the venom, silk and ovary glands from four of the spider species (Table 2). Furthermore, we provide support for phylosymbiosis across this clade of spiders based on our observations of the host phylogeny mirroring the majority of the microbial beta-diversity trees (Fig. 4 and Fig. S4). Phylosymbiosis was also observed across several tissue types from these spiders, including the ovaries.

We evaluated the microbial community diversity for this clade of widow spiders and their specialized tissues. The PCoA beta-diversity plots tend to show a species-specific clustering pattern for these spider microbiome samples. We found a significant clustering of *L. geometricus* samples within the Weighted-UniFrac beta-diversity PCoA plots. We suspect that this clustering indicates limited microbial diversity and is due to the microbiome of *L. geometricus* being dominated primarily by *Candidatus Rhabdochlamydia* a putative intracellular bacteria. *Candidatus Rhabdochlamydia* was observed to be significantly abundant in each set of tissues for *L. geometricus* with ANCOM testing, indicating that this may be a species-specific microbial community member. This limited microbial diversity may be due to *L. geometricus,* a presumed African endemic, being recently introduced to North America (Florida and California) and expanding its range from a small populations [23]. We hypothesize that the low microbial diversity of *L. geometricus* may mirror limited genetic diversity in the host population due to a bottleneck; putatively, bottlenecking the population could have restricted the variation of bacterial diversity that is vertically transmitted [29]. *L. geometricus* sampled for this study were collected from the vicinity of Sarasota, Florida and were inbred. The colonies of the *S. grossa* and *P. tepidariorum* may have been inbred as well, but the original females of these colonies were possibly from more genetically diverse populations. However, based on detecting phylogenetically distinct microbial communities within each spider species’ ovaries, some of these microbial symbionts are possibly vertically transmitted as observed in other systems [8]. This would explain the phylogenetic congruity between microbial diversity and spider phylogeny. The results from PCoA beta-diversity plots also demonstrate that the spiders’ prey items (crickets) have a microbial community distinct from all spider samples (Fig. 1a & c). This suggests that the cricket microbiota does not contribute to the dominant microbial community members within the spider samples and that isolation of the host subject prior to dissections could have helped isolate the signal of non-prey/host specific microbiome from the prey-item “background” microbial community profile.

We were able to identify microbial community members in multiple types of spider tissues and determine which microbial taxa are unique to venom and silk glands. For example, we found that spider species more evolutionarily distant (*P. tepidariorum* and *S. grossa*) to the black widow species (*L. hesperus* and *L. mactans*) had more unique bacterial taxa in their venom and silk glands (Table S4A & S4B). The fewer unique microbial community members in *Latrodectus* venom glands suggest that this toxin-rich environment selected for only a small set (8 genera) of venom tolerant microbiota. *Psychrobacter* and *Variovorax* are two microbial members unique to venom glands that were found in two sets of spider species (*P. tepidariorum* and *L. geometricus* - *Psychrobacter*, *S. grossa* and *L. mactans* - *Variovorax*). *Psychrobacter* is a gram-negative, non-motile coccobacilli that is exceptionally cold-tolerant [30], and has been isolated from the guts of several fish species [31], and detected in the rectums of decomposing swine [32]. *Variovorax* is a genera of gram-negative bacteria found capable of catabolizing toxic and complex compounds and found in water and soil [33]. *Variovorax* has also been determined to be a gut symbiont of beetle larvae (*Holotrichia parallela)* [34], mosquitoes (*Anopheles culicifacies*) [35] and sand flies (*Phlebotomus papatasi*) [36]. Furthermore, our findings of a limited set of microbial community members unique to *Latrodectus* venom glands is supported by recent research that demonstrates the significant and potent anti-microbial properties of venoms isolated from a wide range of spider species and the possibility that venom may elicit a “preservative” effect on prey items [37, 38].

Additional microbial symbionts of other arthropods, some with potential adaptive function, were identified within our spider samples. *Gilliamella* was found across four of the five spider species and was dominant in *L. mactans* and *L. hesperus* samples. Interestingly, *Gilliamella* has been determined to be a major symbiont of bees and was isolated from the guts of *Apis mellifera* [39–41] and multiple *Bombus* species [40–42]. *Spiroplasma* is also a well-characterized bacterial symbiont of many arthropods and was found to dominate the whole *L. hesperus* samples [43–45]. This particular symbiont is thought to provide certain arthropod hosts protection against parasitoid wasps, nematodes and fungal pathogens, but is considered pathogenic in bees (*A. mellifera*) and mosquitoes (*Culex tritaeniorhynchus* and *Aedes sollicitans)* [43]. *Spiroplasma* has also been documented as a symbiont of other spider species [14, 15, 17, 20] and includes spiders within the following families: Gnaphosidae, Lycosidae, Araneidae, Linyohiidae, Tetragnathidae, Scytodidae, and Linyphiidae. Goodacre et al. [17] screened widow spiders for *Spiroplasma* but this symbiont was not observed; however, only three individuals (species not indicated) were evaluated and the authors utilized *S. ixodetis* specific rather than universal 16S PCR primers. Additionally, we detected several other known arthropod symbionts across several spider species (*Bartonella* [36, 46], *Ralstonia* [47], *Acinetobacter* [12, 20, 47], *Delftia* [47], *Rhizobiales* [47, 48], *Sphingomonas* [20, 47], *Propionibacterium* [7], and *Sphingomonadaceae* [47, 49])*.* We also found evidence that several of the microbial community members observed in *P. tepidariorum, S. grossa, L. geometricus* and *L. hesperus* are possibly metabolically active, as transcripts for these symbionts were found in corresponding RNA sequencing datasets (RNA-Seq data was unavailable for *L. mactans*). Although we cannot discount the potential role that husbandry, housing, and acquisition might play in the assembly of these communities, these results support the possibility that the above-mentioned microbial symbionts are viable and functional members of these widow species’ microbiomes. The majority of these concordant RNA transcripts and 16S detected microbiota have also been reported as symbionts within other arthropod microbiomes [47] and include the following taxa: *Bradyrhizobium* in spiders [20], *Burkholderiales* in ants [50], *Methylobacterium* in jewel wasps, mosquitoes, and spiders [4, 20, 51], *Pseudomonas* in mosquitoes, honey bees and other spiders [20, 46, 51], *Sphingomonas* in spiders [20], *Stenotrophomonas* in beetle larvae [34], *Staphylococcus* in jewel wasps [7] and *Wolbachia,* a well-known symbiont of many arthropods*.* It is also possible that some of the *Wolbachia* transcripts identified could have originated from horizontally transferred genes expressed within these spider hosts, as *Wolbachia* and its prophage WO possibly have undergone HGT events with *Latrodectus* species [24]. In such cases it is challenging to separate out host verse microbial transcripts from RNA sequencing data. Additionally, both assays detect *Wolbachia* within the ovary tissues from *L. geometricus.* More interestingly, the sources and locality of the spiders sampled in the RNA sequencing studies were sampled from geographical sites different from those of the spiders collected to generate this study’s 16S dataset (except for *S. grossa*). Even with these different sampling conditions, we are still able to detect multiple species of the same bacterial symbionts between these datasets, indicating these may be species-specific microbiota.

We predicted that we would observe phylosymbiosis across the host phylogeny and we found evidence of this eco-evolutionary pattern of microbial community assembly within these widow spider host species and their respective tissues (except fat tissue). This is supported by the observed congruency of the host phylogeny and multiple microbial beta-diversity dendrograms (Fig. S4). If the microbial communities across these tissues and whole spider species samples were stochastic/ randomly assembled, then we would have expected to observe the dendrograms to not parallel each other, have high Robinson-Foulds Cluster and Matching Cluster measurements and resemble the random pattern / incomplete congruency found between the fat tissue microbial diversity trees (Fig. S4). The non-congruent fat tissue trees could have been due to a comparative lack of host-species-specific tissue specialization. Therefore, each spider species and its specialized tissues appear to have phylogenetically distinct microbial communities that were not specifically acquired by their immediate environment, as our results suggest an “ancestral microbial signal” being retained across the host phylogeny [7, 8]. This “signal” implies that species’ microbial communities are more similar to each other but diverge from when they share their last common ancestor; akin to homology of genetic pathways in a genome that diverge but indicate a common ancestor. Although, we do not know what the genetic sequence of the common ancestor was, much like we cannot know what the shared ancestral microbiome was. The same eco-evolutionary pattern of phylosymbiosis has also been observed across such distant taxa as jewel wasps (*Nasonia*), fruit flies (*Drosophila),* rodents (*Peromyscus, Mus,* and *Neotoma),* mosquitoes, and wild hominids [4, 8, 51, 52]. However, phylosymbiosis of host species’ tissue specific specialization has never been described before to our knowledge. Together with these recent studies of multiple hosts and their microbe interactions, we add additional support for the hypothesis of phylosymbiosis as a biological process.

The hologenomic theory of evolutionary theory is supported through observations of phylosymbiosis of these hosts’ microbiota with the resulting analysis of the ovary tissues, implicating heritability of complex communities of microbes from one generation to the next. However, the genesis of the associated microbiome to the ovaries could also be environmentally acquired or even transient representations of the microbiome. It is important to note that vertical transmission of the spiders’ microbiota is not yet demonstrated, outside of the observations of endosymbionts *Wolbachia* and *Cardinium*, but is also not an obligatory condition for the hologenomic theory of evolution (see Brooks et al. 2016). Specifically, the paralleled host phylogenetic tree and the ovary specific microbial beta-diversity trees (Bray-Curtis and Jaccard) indicate that there are phylogenetically distinct microbial community members present in widow spider ovaries and these symbionts could be vertically transmitted to progeny as the observations were consistent between individuals within a species. We also detected unique microbial community members for each species’ ovary tissues (Table S5A), a counter point to random associations of microbes in a host. Further evidence to this is the presence of the well-documented, heritable, arthropod symbionts. *Wolbachia,* for example, was in each set of ovaries, except for the more distantly related *P. tepidariorum*. *Wolbachia* has been evaluated in past studies for possibly causing sex-ratio distortion in spiders [10, 13]. Sex-biased prevalence of *Wolbachia* (more females infected verses males) was observed in two of the 27 spider species surveyed by Duron et al. [13] (*Meta mengei* and *Tetragnatha montana*), suggesting *Wolbachia’s* involvement in the sex-ratio manipulation of spiders. Research on the dwarf spider (*Oedothorax retusus*) has also indicated that bacterial symbionts play a role in sex-ratio distortion, most significantly by maternally inherited *Wolbachia* [10]. Due to the dwarf spider’s smaller clutch sizes, it is speculated that the mechanism for this phenomena is the male-killing of embryos [10]. Other known bacterial symbionts observed in our spider samples include *Cardinium,* which was noted by Duron et al. [13] to be transmitted from mother to progeny within marbled cellar spiders (*Holocnemus pluchei*); however, this symbiont does not seem to skew sex-ratios or manipulate reproduction as observed in other arthropods [2, 13, 16]. *Cardinium* was also observed to be a major, dominant symbiont in three other spider species *H. graminicola*, *U. insecticeps,* and *A. difficilis* [20]*.* However, we did not detect *Cardinium* in these selected widow related spider species.

## Conclusions

In conclusion, we provide evidence for phylosymbiosis across black widow related spiders in the Theridiidae Family. Our results support the phylosymbiotic pattern of microbial communities across whole spider samples and several tissue sample sets (cephalothorax, venom glands, silk glands and ovaries) based on congruency between the widow phylogeny and multiple microbial beta-diversity dendrograms. This specialization of tissues and microbiomes within a species represents a unique facet of phylosymbiotic evolution not previously described - tissue tropic phylosymbiosis. We also provide evidence for possible maternal-offspring transfer of phylogenetically distinct microbial communities based on our phylosymbiosis analyses and characterization of the microbiota from spider hosts’ ovary tissues. These observations suggest that the diversity of symbiotic microbial communities within and across spider species is hypothetically, in part, vertically inherited. Thereby, the host-microbiome association is putatively evolving in response to host speciation and has had the potential to shape host evolution.

## Methods

### Sample acquisition

Adult female spiders were acquired from Spider Pharm (Yarnell, AZ). These spiders include the following species: *Parasteatoda tepidariorum*, *Steatoda grossa, Latrodectus geometricus, Latrodectus hesperus, Latrodectus mactans.* Female spiders were confirmed to have reached adulthood by examining the epigynum prior to dissections. Each spider was treated / housed in identical conditions and fed crickets (Vita-Bug, common brown cricket – Timberline Fisheries, Marion, IL) from the same batch/lot whenever possible.

### Last feeding and environmental isolation

At least six adult female spiders of each species were fed one cricket on the same day. After 24 h − 32 h each spider was transferred from the feeding vial into a sterile housing container. Each spider was isolated in this sterile environment for 7–8 days, without any additional feedings. The goal of this isolation procedure was to “starve” spiders to reduce the signal/ background microbial community of the spider’s prey.

Twelve crickets were also included in this study to determine background / food sourced microbial community members. Prior to the first round of spider dissections, crickets from the same batch underwent the same isolation conditions as the spiders. An additional three crickets were used during the second round of spider dissections (for second sequencing run) to determine the cricket microbiome at the time of the respective spiders’ last feeding. These cricket controls were euthanized on the same day as the last feeding and stored immediately at − 80 °C.

### Aseptic spider tissue dissections

Prior to tissue dissections, forceps, wash containers/ beakers, and dissection buffer (SSC buffer) were autoclaved and sterilized. The microscope and surrounding area were cleaned with 10% bleach and 70% ethanol. After each dissection, the forceps were sterilized with a bleach wash, ethanol wash, followed by a sterile PCR water (VWR) rinse prior to the next spider dissection. Each spider underwent surface sterilization to remove possible environmental contaminants with a 10% bleach soak for 1 min followed by two separate washes in sterile PCR water for 1 min each (adapted from Brooks, A.W., et al. [8]). Three to four individual spiders of each species had the following tissues dissected in an aseptic manner [53]: venom glands [54], cephalothorax (without chelicerae), silk glands, ovary, and fat / mid-gut region.

Each tissue was rinsed with sterile PCR-grade water prior to collection in a sterile 1.5 mL microfuge tube. Three to four individual spiders of each species (whole, no dissection) were also surface sterilized prior to transfer to a sterile 1.5 mL microfuge tube. All cricket samples were transferred to 1.5 mL tubes without surface sterilization. All samples and an aliquot of SSC buffer were frozen in liquid nitrogen and then stored at − 80 °C until DNA extraction.

### DNA extractions

DNA was extracted from each spider (3 per each species), spider tissue (3 sets per species), cricket and negative controls (SSC buffer and DNA extraction controls (reagents only) using Qiagen’s QiaAMP DNA Mini kits. We utilized Qiagen’s protocol with the following specifications: liquid nitrogen to freeze samples prior to homogenizing with a motorized pestle, 1.5 h lysis (vortexing every 20–30 min), a centrifugation step for 30 s at 6000 g prior to transferring sample lysates to their respective columns, and two elution steps (except for venom glands) - each with 5 min room temperature incubations. Prior to the DNA extractions of large whole spiders (*Latrodectus* species), each individual spider was divided in half with sterilized razor blades and forceps and the mass of each half was measured. DNA was extracted from both halves separately to avoid overloading the columns. Eluted DNA was combined in equal ratio based off of the pre-processed weight. Each sample type had an optimal elution volume, based on the size of the tissue or if a whole sample (spider or cricket). These elution volumes were as follows: whole spider = 400ul, cricket = 200ul, cephalothorax = 200ul, venom glands = 50ul, ovaries = 100ul, silk glands = 100ul, fat = 100ul.

DNA extractions were performed aseptically, with re-directed airflow, and while wearing a facial mask to reduce the risk contaminating the samples with exhaled bacteria. The extracted DNA was then quantified with ThermoFisher’s Quant-iT dsDNA High Sensitivity kit.

### 16S rRNA gene amplicon library preparation and sequencing

The standard methods for taxonomic classification of bacteria within a microbial community utilize the small ribosomal subunit (16S rRNA) gene. The 16S rRNA gene contains nine hyper-variable regions of various lengths. The variable regions with highest confidence of identifying bacteria down to the genus and species level to date are the V1-V2 and V1-V3 regions [55, 56]. The V1-V2 region was selected for this study because it is ~ 310 bp long and the appropriate length for higher quality paired-end sequencing with the Illumina MiSeq. Furthermore, utilizing the V1-V2 target is 90% accurate for identifying bacteria at the species level and 92% accurate at the genus level [55]. Prior to commencing this study, over twenty spider samples (whole and tissue) were used to test different sets of universal polymerase chain reaction (PCR) primers that target the V1-V2 region (27F-338R) and V3-V4 region (338F-786R). The 27F (5′-AGAGTTTGATCMTGGCTCA-3′ – slightly modified from Brooks et al.) and 338R (5′-GCTGCCTCCCGTAGGAGT-3′) universal 16S primers amplified the expected ~ 310 bp V1-V2 region from < 90% of test samples [8].

The V1 and V2 variable regions of the 16S rRNA gene were amplified from the extracted DNA and mock community DNA control (ZymoBIOMICS™ Microbial Community Standard from Zymo Research) utilizing universal PCR primers, 27F and 338R [8]. PCR was completed in a two-step process (PCR-1 and PCR-2) in order to yield significant PCR product with a unique molecular barcode for each sample’s 16S amplicons [49, 57, 58]. We designed custom primers containing V1 and V2 regions following the 16S primer design protocol by Kozich and Schloss [59], where the 27F and 338R primers contain a unique 8 bp barcode on each primer, a short Linker/ Pad sequence and the appropriate Illumina adaptor sequence (i5 or i7) (see Table S1 for list of primer sequences). Preliminary data showed that two-step PCR yielded significantly better results (consistent visible bands from gel electrophoresis) than nested-PCR. These multi-step PCR processes were also compared with single step PCR, where single-step PCR resulted in inconsistent and/ or low amplification of the spider microbiome DNA samples.

Extracted DNA from each sample and all negative controls (SCC buffer, Negative Extraction Controls, and PCR-water (non-template control) were run through one round of PCR-1 using a 12.5ul reaction with Q5 high fidelity master-mix (New England BioLabs, Inc.) with the following cycling conditions: 98 °C for 30 s, 25 cycles of 98 °C for 30 s (denature), 50 °C for 30 s (anneal), and 72 °C for 30 s (extension), with a final extension step at 72 °C for 10 min and end hold at 4 °C. PCR-2 included 2-3ul of PCR-1 product as template DNA. Four PCR-2 replicate 25ul reactions, using Q5 high fidelity master-mix, were generated per sample (3 with sample PCR-1 product and 1 as a non-template control). The conditions for PCR-2 were as follows: 98 °C for 30 s, 15 cycles of 98 °C for 30 s (denature), 50 °C for 30 s (anneal), and 72 °C for 30 s (extension), with a final extension step at 72 °C for 10 min and end hold at 4 °C. Each set of PCR-2 product replicates were combined per sample and purified with AMPure XP beads in a 1.8X bead-to-product ratio [60]. Each purified sample was then normalized to the same molar mass using Qubit Fluorometric Quantification (ThermoFisher Scientific). Two final normalized, pooled sample libraries and custom sequencing primers (Table S2) were sent to Cornell University’s Genomics Facility (according to their protocol) for two runs of paired-end sequencing (2 × 250 bp), with a 10% PhiX spike in, on an Illumina MiSeq following the Kozich and Schloss MiSeq protocol [59]. The concentration of sequencing primers was doubled for the second round of sequencing in order to increase the number of high-quality reads.

### Microbial community data analysis

#### Pre-processing of sequences and initial quality control

The Quantitative Insights into Microbial Ecology (QIIME) program was utilized for pre-processing sequencing reads and microbial community analyses [61]. QIIME 1 was used to add barcodes to the read files (merge_bcs_reads.py), extract barcode sequences from the reads (extract_barcodes.py), join overlapping paired-end reads (join_paired_ends.py), and lastly demultiplex the joined reads according to their respective barcodes and sample IDs (split_libraries_fastq.py). After joining the paired-end reads (un-joined reads were removed from downstream analyses), the demultiplexing script also passes reads through quality filtering (reads < Q20 were removed from the dataset). A total of 896,429 reads out of 3,578,685 passed initial quality filtering for the first run and 5,172,436 reads out of 10,211,041 passed from the second sequencing run (large percentage of reads lost to PhiX spike-in and joining-step).

#### Sequence Dereplication, chimera checking, OTU picking, and taxonomy assignments with QIIME

The resulting joined, demultiplexed and high quality reads from each MiSeq run are contained in their run-specific seqs.fna output file and were imported into QIIME 2 [62]. Each set of sequences were dereplicated and de novo chimera-checked via the VSEARCH plug-in tool [63]. Chimeric reads (i.e. PCR artifacts/biases from parental strands acting as primers during PCR – hybrid 16S sequences from two different species of bacteria that artificially affect diversity estimates) were removed from the dataset to reduce the impact of PCR errors prior to Operational Taxonomic Unit (OTU) clustering and diversity analyses [8, 64]. The feature-table plug-in was then utilized to merge the two sets of resulting high quality sequences (merge-seqs option) and feature/ OTU tables (merge option) from each of the runs together for downstream analyses. The resulting feature table and sequences files were run through open-reference OTU picking with VSEARCH utilizing SILVA’s 16S QIIME formatted database (release 128–99% identity sequences) at a 99% identity threshold for clustering [63, 65–67]. Low abundance OTUs were filtered out from the resulting feature table, where OTUs with a frequency of less than 10 sequences across all samples were removed. Taxonomy assignments were also generated with SILVA rRNA Database (release 128–99% consensus taxonomy, 7 levels) by extracting out only the 16S V1-V2 regions that correspond to the 27F – 338R primers used for sample library preparation – truncation length of 500 bp (feature-classifier plug-in, extract-reads option) [68]. QIIME 2’s Naïve Bayes classifier was trained to these extracted V1-V2 reference sequences with the feature-classifier plug-in (fit-classifier-naive-bayes). We then utilized this V1-V2 trained classifier set to complete taxonomic assignments with the classify-sklearn feature-classifier plug-in option. After assigning taxonomy to the OTUs, taxa plots were generated with the taxa plug-in (barplot command) and all OTUs that were classified as unassigned, chloroplasts, and / or mitochondria were filtered out from the feature/ OTU table (taxa filter-table command) and representative sequences. Each of the tissue sample groups feature tables were summarized (feature-table summarize command), taxa barplots generated and reviewed via QIIME 2 View. The resulting level 3 (class) and level-6 (genera) csv files were analyzed via R with the following packages: dplyr, tidyr, stringr, and digest [69–73]. The OTUs that made up at least 2% or greater relative abundance across each tissue set was used to generate barplots with ggplot 2 [74].

Alignments were completed on the set of representative sequences with MAFFT (Alignment MAFFT plug-in) [75] and the unconserved, highly gapped columns in these aligned sequences were masked with the alignment plug-in, mask command [76]. A phylogenetic tree was then generated with the FastTree 2 tool (Phylogeny plug-in) using a Maximum-Likelihood method [77]. The resulting tree was then midpoint rooted (Phylogeny plug-in, midpoint-root option) and utilized for downstream beta-diversity analyses.

#### Mock community standard and quality assurance measures

The data from the first sequencing run underwent quality control measures (Table S3) to ensure the sample library preparation steps and sequencing performed as expected prior to moving forward with sample library preparation and second sequencing run for the majority of the spider samples. This step was also completed to determine if the OTU clustering threshold of 99% was appropriate for the data analysis pipeline, in order to reduce the potential of erroneously generating OTUs. Quality control assessments were completed by comparing the percentage of each mock community member present within the first sequencing run dataset to the theoretical/ expected mock community composition as provided by the manufacturer, Zymo Research. The preliminary results of the mock community analysis indicated that the sample library preparation method and sequencing specifications were appropriate and accurately measured the mock community composition. Furthermore, one of the whole *P. tepidariorum* replicate samples (note: total of 3 replicates per sample) from the first sequencing run was repeated through the library preparation process and second MiSeq run as a positive control to test the differences between runs.

#### Diversity analyses

After OTU clustering, taxonomy assignments, taxonomy-based filtering, and 16S rRNA gene alignments, the feature/ OTU table was rarefied based on the depths of coverage per sample type. A depth of 7032 randomized sequences per sample was selected for the core-metrics beta-diversity analyses (prior to any grouping of replicate samples) based on this depth of coverage encompassed all but one spider tissue sample (this sample contained a very high percentage of chloroplast related OTUs, which were filtered out in upstream data processing steps) and the alpha-diversity results from running the diversity plug-in, alpha-rarefaction command run on spider samples (Fig. S1A & B, depth range of 18–18,832 sequences). QIIME 2’s diversity plug-in core-metrics-phylogenetic command was run on all the filtered samples (crickets included), each set of tissue types, and then on only the spider samples (crickets removed); in order to best resolve the resulting UniFrac distance matrix derived EMPeror PCoA plots [78]. Box-plots were generated, along with group significance statistical testing (using the QIIME diversity alpha-group-significance which is a Kruskal-Wallis one-way analysis of variance), from each resulting alpha-diversity test vector file utilizing the diversity plug-in alpha-group-significance command [79]. Statistical analyses were completed to determine significant changes in the abundances of the microbial community members between and across samples from each tissue type with ANCOM testing [80]. Sample (biological) replicates were then grouped with the feature-table group command (mean-ceiling, i.e. average frequency of each OTU across sample replicates) by base sample type - whole spiders, cephalothorax, venom glands, ovaries, silk glands and fat tissue (one grouped table per tissue type – resulting in 6 grouped tables - only these grouped tables, not individual replicate samples, were used for the beta-diversity dendrograms for phylosymbiosis analyses). A master grouped feature table was also generated for all the spider samples – grouping each replicate sample by the mean-ceiling option. Each of these grouped feature tables were summarized (feature-table summarize command), had taxa barplots generated and each were reviewed via QIIME 2 View. Each grouped tissue type feature table was then passed though the diversity beta-rarefaction command with a selected depth of coverage determined by the lowest sample sequence/ feature count per each set of grouped tissue samples (Whole Spiders = 14,509, Cephalothorax = 20,799, Venom = 20,826, Ovaries = 28,629, Silk = 28,628, Fat = 17,074) for the following beta-diversity tests: Bray-Curtis, Jaccard, Unweighed UniFrac [81] and Weighted UniFrac [78]. Furthermore, a PERMANOVA test was conducted to look at pairwise differences in beta diversity (Bray-Curtis Distances) between species or tissues was conducted as defined by the conditions of the ADONIS function within QIIME (Supplemental Table 8) [82].

#### RNA sequencing Metatranscriptome analysis

Publicly available RNA sequencing data from previous research completed by Garb *et. al*, specific to silk, venom, and ovary glands, were acquired from NCBI for the following species: *P. tepidariorum*, *S. grossa*, *L. geometricus*, and *L. hesperus.* The collection sites for these spiders are as follows: *P. tepidariorum* (silk, venom, and ovary) - Cologne, Germany, out-bred with *P. tepidariorum* spiders from Spider Pharm/ Arizona, United States, *L. hesperus* (silk, venom and ovary) - Riverside County, California, United States, *L. geometricus* (silk, venom, and ovary) - San Diego County, California, United States. *S. grossa* was obtained directly from Spider Pharm (similar to samples used for 16S sequencing). The following SRA files were downloaded from NCBI’s SRA database: SRR1219665, SRR1824489, SRR5131057, SRR5131058, SRR5285094, SRR5285095, SRR5285096, SRR5285099, SRR5285100, SRR5285114, SRR5285115, SRR5285118, SRR5285121, SRR5285122, SRR5285123, SRR5285135, SRR5285136, SRR5285138, SRR5285141 and SRR5285142 [21, 53, 83, 84]. Fastq files were extracted from each SRA file utilizing the SRAToolkit, fastq-dump command [85]. The extracted fastq files were run through FastQC and then the adaptors and poor quality bases were trimmed with Trimmomatic [86, 87]. The Trimmomatic parameters utilized for trimming the raw reads are as follows: crop_length = total read length – 1 (between 75 and 100 depending on library), seed_mismatches = 2, paired_end_seed_score = 30, min_adapter_length = 2, keepBothReads = true, sliding_window_size = 10, sliding_window_minimum_average_phred_score = 15, min_length_to_keep_reads = 36, trimmomatic_threads = 8 [86, 87]. Corresponding Read 1 and Read 2 sequences for each species silk, venom or ovary glands were concatenated, gzipped and uploaded to CosmosID for identification of any possible microbial transcripts / sequences, as Cosmos ID is capable of uploading and analyzing raw or processed read data [88]. CosmosID takes raw, unassembled reads and matches them to the GenBook® database, utilizing statisticial and and computational methods [89].

### Phylosymbiosis analyses

The following analysis methods were used to determine if there is evidence of phylosymbiosis across the selected widow related spider phylogeny as described by Brooks et al. [8]. Mitochondrial COI gene sequences were obtained from a previous study, where a 428–659 bp fragment was sequenced for each spider species represented in this study [22]. The five resulting mtCOI sequences were aligned with ClustalX v2.1 in multiple alignment mode [90]. The aligned sequences were exported in Newick format and run through jModeltest (2.1.10 v20160303) to determine the best substitution model for generating a widow spider phylogenetic tree [27, 91]. The aligned sequences were then utilized to generate a Maximum-Likelihood tree with RAxML v8.2.11 (GTR + γ substitution model and 10,000 iterations) [26]. The resulting tree was viewed and rooted with the *P. tepidariorum* branch utilizing FigTree v1.4.3 [92]. The resulting widow phylogenetic tree and each of the tissue specific and whole spider microbial beta-diversity dendrograms were tested for congruency by utilizing Robinson-Foulds Cluster and Matching Cluster tests to compute the distances (dissimilarity) between the host phylogeny and each microbial beta-diversity tree (both trees rooted) [8, 28].

## Supplementary information

**Additional file 1: Figure S1.** Study Design Flowchart. Overview of methods utilized to isolate, evaluate, and compare the microbiomes of our host spider species and their tissue samples.

**Additional file 2: Table S1.** PCR 16S rRNA gene V1-V2 Primer Sequences.

**Additional file 3: Table S2.** Sequencing Primers.

**Additional file 4: Table S3.** Comparative mock community composition.

**Additional file 5: Figure S2.** QIIME 2 Microbial Alpha-Diversity Plots. Alpha-Rarefaction results for Faith-PD (a), Observed OTUs (b), and Shannon Index (c) with Depth of Coverage at 8000 rarified sequences.

**Additional file 6: Figure S3.** Taxa Plots per Tissue Type – Level 3, Class. Major microbial constituents by class (> 2%) within each whole spider and tissue sample for each species (replicates grouped via mean-ceiling). Sample types as follows: Whole Spiders, Cephalothorax, Venom Glands, Ovary, Silk Glands, and Fat Tissue.

**Additional file 7: Figure S4.** QIIME 2 Alpha Diversity Group Significance Testing. Box plots of alphadiversity tests grouped by base sample type. Overall Kruskal-Wallis *p*-value = 0.00028 (for all groups). (a) Observed OTUs, (b) Faith Phylogenetic-Diversity Index, (c) Shannon Index, (d) Pielou Evenness Index.

**Additional file 8: Tables S4.** Listings of venom gland (a) and silk gland (b) specific microbiota.

**Additional file 9: Table S5.** Listings of ovary gland (a) and fat tissue (b) specific microbiota.

**Additional file 10: Figure S4.** Phylosymbiosis between Widow Phylogeny and Microbiota Dendrogram Relationships. Congruency between widow related spider phylogeny and beta-diversity dendrograms was measured via Robinson-Foulds Cluster (R-F Cluster) metric, where a value of 0 = complete congruence and 2 = incomplete incongruence. Matching Cluster (MC) Metric also accounts for incongruency between closely related branches of rooted trees and a value of 0 also indicates complete congruence between compared trees. Pink asterisks are utilized to denote phylosymbiotic relationships between the widow phylogeny and microbial dendrogram.

**Additional file 11: Table S6.** Microbial transcripts detected in RNA sequencing datasets from silk, venom and/ or ovary glands.

## Data Availability

Custom scripts generated to analyze the QIIME 2 output files are locate on github at the following website: https://github.com/SaraJeanne08/Microbiome_Related 16S amplicon sequencing reads from both MiSeq 2 × 250 bp runs are deposited into the Dryad Digital Repository [91]: 10.5061/dryad.8c3k3t9

## Abbreviations

NGS: Next Generation Sequencing
16S: Small Ribosomal Subunit
PCR: Polymerase Chain Reaction
OTU: Operational Taxonomic Units
PCoA: Principle Coordinates Analysis
AIC: Akaike Information Criterion
BIC: Bayesian Information Criterion
GTR + γ: General Time Reversible – Gamma Distribution
SSC buffer: Saline-Sodium Citrate buffer
