# Supplementary material for: Spider phylosymbiosis: divergence of widow spider species and their tissues’ microbiomes
Source: BMC Evol Biol. 2020 Aug 18;20:104. doi: 10.1186/s12862-020-01664-x (PMC7433143; doi:10.1186/s12862-020-01664-x)
Supplement: Supplementary file 1 — Additional file 1: Figure S1. Study Design Flowchart. Overview of methods utilized to isolate, evaluate, and compare the microbiomes of our host spider species and their tissue samples. [file 12862_2020_1664_MOESM1_ESM.pdf]

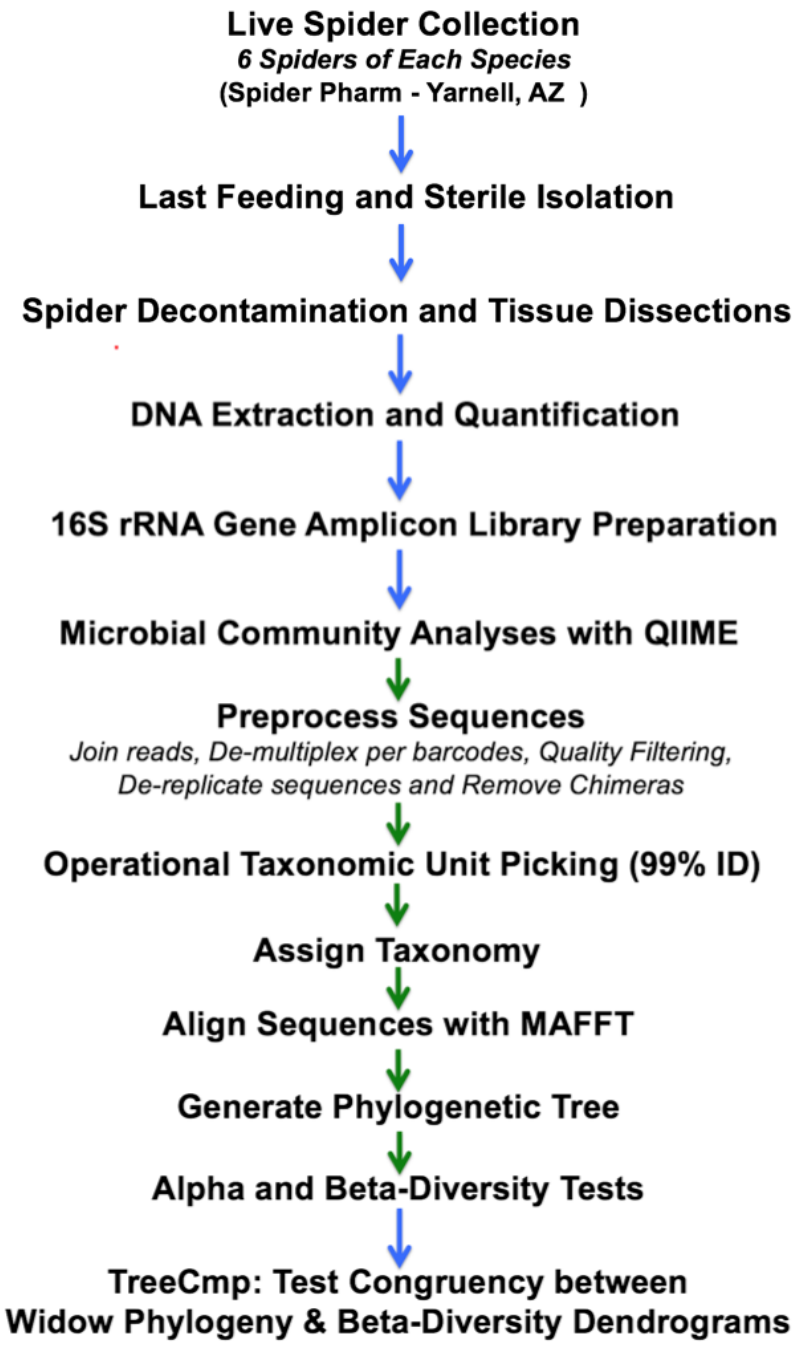

Figure S1. Study Design Flowchart. Overview of methods utilized to isolate, evaluate, and compare the microbiomes of our host spider species and their tissue samples.
