# Supplementary material for: Spider phylosymbiosis: divergence of widow spider species and their tissues’ microbiomes
Source: BMC Evol Biol. 2020 Aug 18;20:104. doi: 10.1186/s12862-020-01664-x (PMC7433143; doi:10.1186/s12862-020-01664-x)
Supplement: Supplementary file 2 — Additional file 2: Table S1. PCR 16S rRNA gene V1-V2 Primer Sequences. [file 12862_2020_1664_MOESM2_ESM.pdf]

# Table S1. PCR 16S rRNA gene V1-V2 Primer Sequences.

| Primer ID | Sequence (3'-5') | Illumina Adaptor | Forward or Reverse | PCR-1 or PCR-2 |
|---|---|---|---|---|
| **16sGenV1F** | AGAGTTTGATCMTGGCTCA | n/a | Forward | PCR-1 |
| **16sGenV2R** | GCTGCCTCCCGTAGGAGT | n/a | Reverse | PCR-1 |
| **v1.SC501** | AATGATACGGCGACCACCGAGATCTACACACGACGTGTATGGTAATTTG AGAGTTTGATCMTGGCTCA | i5 | Forward | PCR-2 |
| **v1.SC502** | AATGATACGGCGACCACCGAGATCTACACATATACACTATGGTAATTTG AGAGTTTGATCMTGGCTCA | i5 | Forward | PCR-2 |
| **v1.SC503** | AATGATACGGCGACCACCGAGATCTACACCGTCGCTATATGGTAATTTG AGAGTTTGATCMTGGCTCA | i5 | Forward | PCR-2 |
| **v1.SC504** | AATGATACGGCGACCACCGAGATCTACACCTGCGTGTTATGGTAATTTG AGAGTTTGATCMTGGCTCA | i5 | Forward | PCR-2 |
| **v1.SC505** | AATGATACGGCGACCACCGAGATCTACACTCATCGAGTATGGTAATTTG AGAGTTTGATCMTGGCTCA | i5 | Forward | PCR-2 |
| **v1.SC506** | AATGATACGGCGACCACCGAGATCTACACCGTGAGTGTATGGTAATTTG AGAGTTTGATCMTGGCTCA | i5 | Forward | PCR-2 |
| **v1.SC507** | AATGATACGGCGACCACCGAGATCTACACGGATATCTTATGGTAATTTG AGAGTTTGATCMTGGCTCA | i5 | Forward | PCR-2 |
| **v1.SC508** | AATGATACGGCGACCACCGAGATCTACACTAGTGTAGTATGGTAATTTG AGAGTTTGATCMTGGCTCA | i5 | Forward | PCR-2 |
| **v2.SC701** | CAAGCAGAAGACGGCATACGAGATACCTACTGAGTCAGTCAGGGGCTG CCTCCCGTAGGAGT | i7 | Reverse | PCR-2 |
| **v2.SC702** | CAAGCAGAAGACGGCATACGAGATAGCGCTATAGTCAGTCAGGGGCTG CCTCCCGTAGGAGT | i7 | Reverse | PCR-2 |
| **v2.SC703** | CAAGCAGAAGACGGCATACGAGATAGTCTAGAAGTCAGTCAGGGGCTG CCTCCCGTAGGAGT | i7 | Reverse | PCR-2 |
| **v2.SC704** | CAAGCAGAAGACGGCATACGAGATCAGTGAGTAGTCAGTCAGGGGCTG CCTCCCGTAGGAGT | i7 | Reverse | PCR-2 |
| **v2.SC705** | CAAGCAGAAGACGGCATACGAGATCGTACTCAAGTCAGTCAGGGGCTG CCTCCCGTAGGAGT | i7 | Reverse | PCR-2 |
| **v2.SC706** | CAAGCAGAAGACGGCATACGAGATCTACGCAGAGTCAGTCAGGGGCTG CCTCCCGTAGGAGT | i7 | Reverse | PCR-2 |
| **v2.SC707** | CAAGCAGAAGACGGCATACGAGATGGAGACTAAGTCAGTCAGGGGCTG CCTCCCGTAGGAGT | i7 | Reverse | PCR-2 |
| **v2.SC708** | CAAGCAGAAGACGGCATACGAGATGGTATGCTAGTCAGTCAGGGGCTG CCTCCCGTAGGAGT | i7 | Reverse | PCR-2 |
| **v2.SC709** | CAAGCAGAAGACGGCATACGAGATGTATGACGAGTCAGTCAGGGGCTG CCTCCCGTAGGAGT | i7 | Reverse | PCR-2 |
| **v2.SC710** | CAAGCAGAAGACGGCATACGAGATTAGACTGAAGTCAGTCAGGGGCTG CCTCCCGTAGGAGT | i7 | Reverse | PCR-2 |
| **v2.SC711** | CAAGCAGAAGACGGCATACGAGATTCACGATGAGTCAGTCAGGGGCTG CCTCCCGTAGGAGT | i7 | Reverse | PCR-2 |
| **v2.SC712** | CAAGCAGAAGACGGCATACGAGATTCGAGCTCAGTCAGTCAGGGGCTG CCTCCCGTAGGAGT | i7 | Reverse | PCR-2 |
