# Supplementary material for: Spider phylosymbiosis: divergence of widow spider species and their tissues’ microbiomes
Source: BMC Evol Biol. 2020 Aug 18;20:104. doi: 10.1186/s12862-020-01664-x (PMC7433143; doi:10.1186/s12862-020-01664-x)
Supplement: Supplementary file 3 — Additional file 3: Table S2. Sequencing Primers. [file 12862_2020_1664_MOESM3_ESM.pdf]

Table S2. Sequencing Primers.

Primer ID:	Sequence:
**Read 1**	TATGGTAATTTGAGAGTTTGATCMTGGCTCA
**Read 2**	AGTCAGTCAGGGGCTGCCTCCCGTAGGAGT
**Index Read**	ACTCCTACGGGAGGCAGCCCCTGACTGACT
