# Supplementary material for: Spider phylosymbiosis: divergence of widow spider species and their tissues’ microbiomes
Source: BMC Evol Biol. 2020 Aug 18;20:104. doi: 10.1186/s12862-020-01664-x (PMC7433143; doi:10.1186/s12862-020-01664-x)
Supplement: Supplementary file 4 — Additional file 4: Table S3. Comparative mock community composition. [file 12862_2020_1664_MOESM4_ESM.pdf]

**Table S3. Comparative mock community composition.**

| Species | Zymo | Zymo | Study QC Measurements |
|---|---|---|---|
| | Theoretical Composition (%) | Measured Composition (%) | Measured Composition (%) |
| | Genomic DNA | Genomic DNA * | Genomic DNA ** |
| *Pseudomonas aeruginosa* | 12 | 14.7 | 11.8 |
| *Escherichia coli* | 12 | 12.6 | 15.2 |
| *Salmonella enterica* | 12 | 12.1 | 15.16 |
| *Lactobacillus fermentum* | 12 | 9.5 | 13.2 |
| *Enterococcus faecalis* | 12 | 12.6 | 8.1 |
| *Staphylococcus aureus* | 12 | 10.2 | 10 |
| *Listeria monocytogenes* | 12 | 12.6 | 9.1 |
| *Bacillus subtillis* | 12 | 12.3 | 16.74 |
| *Saccharomyces cerevisiae* | 2 | 1.9 | n/a - 0 |
| *Cryptococcus neoformans* | 2 | 1.5 | n/a - 0 |

** Shotgun sequencing was performed using the Illumina® MiSeq™ (2x150bp) and with sequencing library prepared with Kapa HyperPlus. The composition in terms of genomic DNA abundance was calculated by summarizing the raw sequencing reads mapped to the genome of each strain.*

*** 16S rRNA Amplicon - Illumina MiSeq (2x 250bp) and Sequencing Library prepared with Schloss Primers - 2 step PCR approach and Sequences at the Genomics Facility at Cornell University.*
