# Supplementary material for: Spider phylosymbiosis: divergence of widow spider species and their tissues’ microbiomes
Source: BMC Evol Biol. 2020 Aug 18;20:104. doi: 10.1186/s12862-020-01664-x (PMC7433143; doi:10.1186/s12862-020-01664-x)
Supplement: Supplementary file 5 — Additional file 5: Figure S2. QIIME 2 Microbial Alpha-Diversity Plots. Alpha-Rarefaction results for Faith-PD (a), Observed OTUs (b), and Shannon Index (c) with Depth of Coverage at 8000 rarified sequences. [file 12862_2020_1664_MOESM5_ESM.pdf]

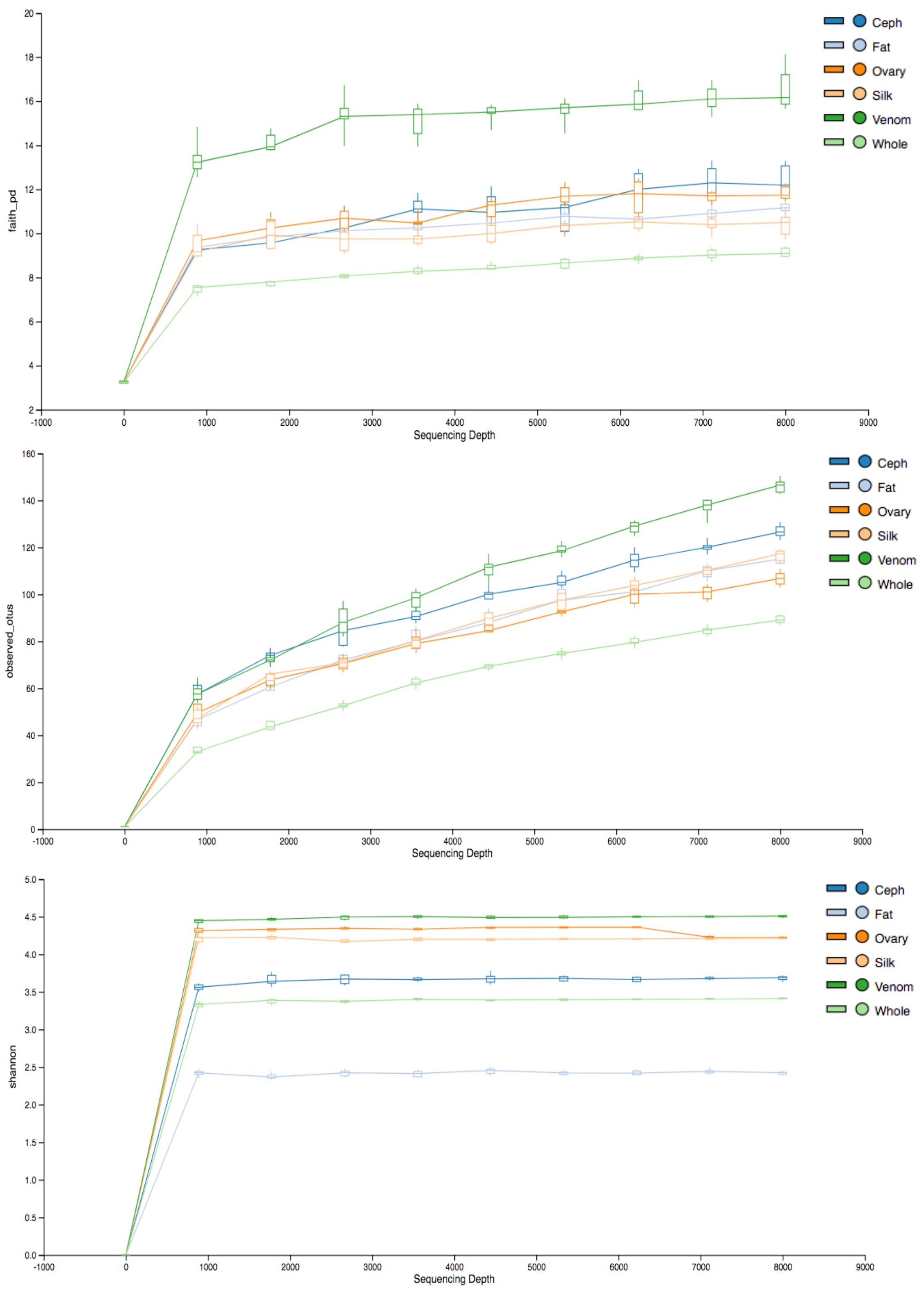

Figure S2. QIIME 2 Microbial Alpha-Diversity Plots. Alpha-Rarefaction results for Faith-PD (a), Observed OTUs (b), and Shannon Index (c) with Depth of Coverage at 8,000 rarified sequences.
