# Supplementary material for: Spider phylosymbiosis: divergence of widow spider species and their tissues’ microbiomes
Source: BMC Evol Biol. 2020 Aug 18;20:104. doi: 10.1186/s12862-020-01664-x (PMC7433143; doi:10.1186/s12862-020-01664-x)
Supplement: Supplementary file 6 — Additional file 6: Figure S3. Taxa Plots per Tissue Type – Level 3, Class. Major microbial constituents by class (> 2%) within each whole spider and tissue sample for each species (replicates grouped via mean-ceiling). Sample types as follows: Whole Spiders, Cephalothorax, Venom Glands, Ovary, Silk Glands, and Fat Tissue. [file 12862_2020_1664_MOESM6_ESM.pdf]

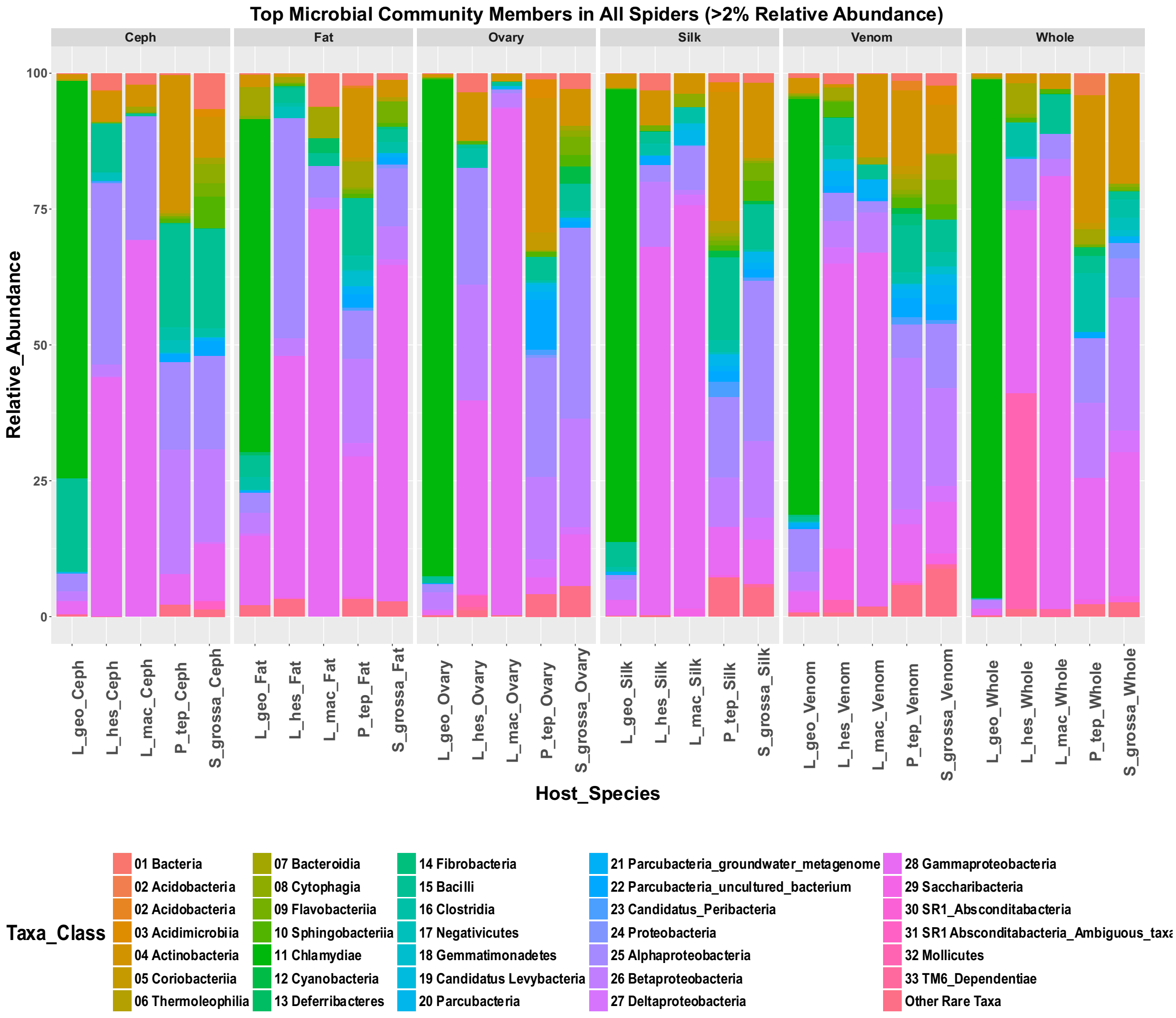

Figure S3. Taxa Plots per Tissue Type – Level 3, Class. Major microbial constituents by class (>2%) within each whole spider and tissue sample for each species (replicates grouped via mean-ceiling). Sample types as follows: Whole Spiders, Cephalothorax, Venom Glands, Ovary, Silk Glands, and Fat Tissue.
