# Supplementary material for: Spider phylosymbiosis: divergence of widow spider species and their tissues’ microbiomes
Source: BMC Evol Biol. 2020 Aug 18;20:104. doi: 10.1186/s12862-020-01664-x (PMC7433143; doi:10.1186/s12862-020-01664-x)
Supplement: Supplementary file 7 — Additional file 7: Figure S4. QIIME 2 Alpha Diversity Group Significance Testing. Box plots of alphadiversity tests grouped by base sample type. Overall Kruskal-Wallis p-value = 0.00028 (for all groups). (a) Observed OTUs, (b) Faith Phylogenetic-Diversity Index, (c) Shannon Index, (d) Pielou Evenness Index. [file 12862_2020_1664_MOESM7_ESM.pdf]

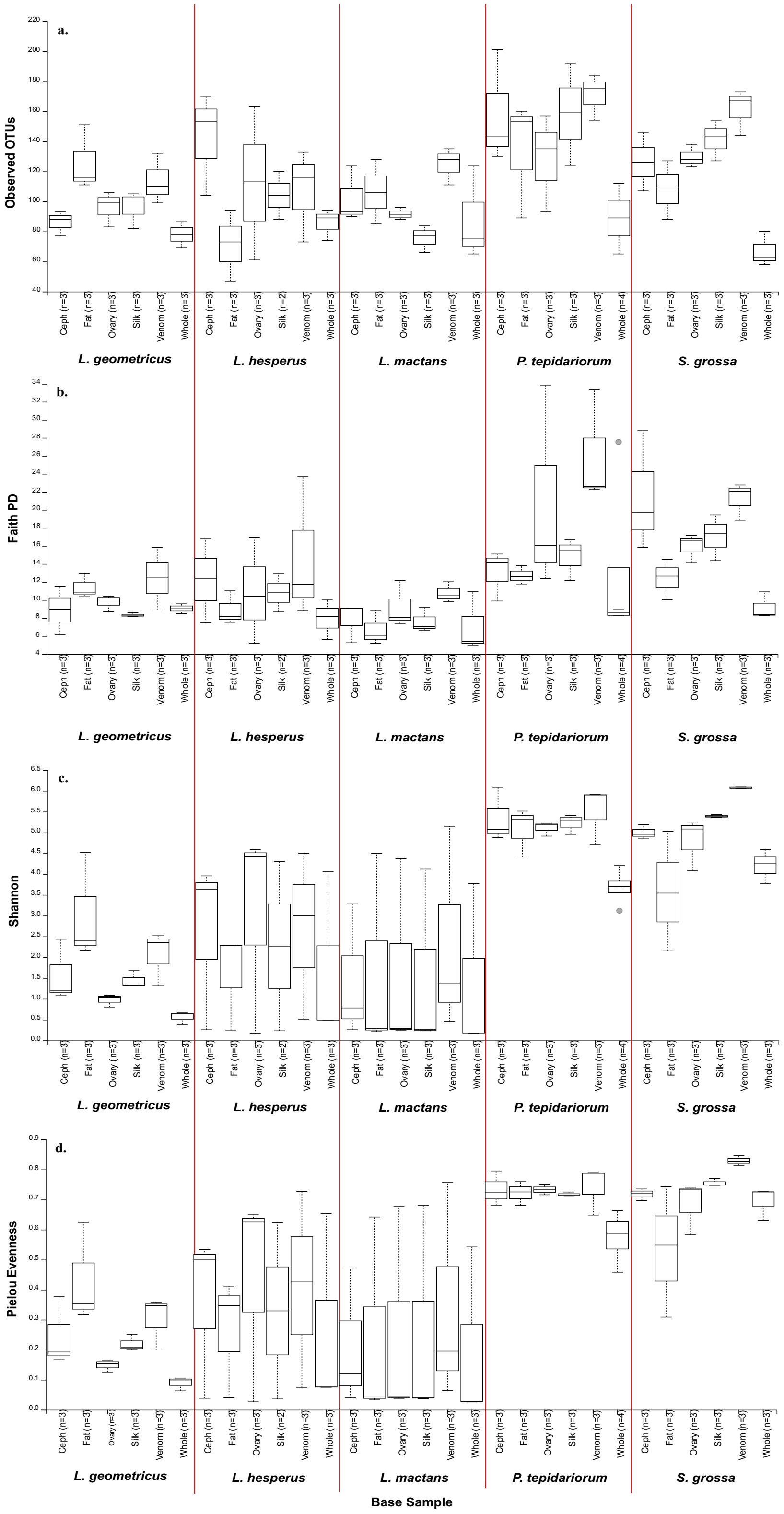

Figure S4. QIIME 2 Alpha Diversity Group Significance Testing. Box plots of alpha-diversity tests grouped by base sample type. Overall Kruskal-Wallis p-value = 0.00028 (for all groups). (a) Observed OTUs, (b) Faith Phylogenetic-Diversity Index, (c) Shannon Index, (d) Pielou Evenness Index.
