# Supplementary material for: Spider phylosymbiosis: divergence of widow spider species and their tissues’ microbiomes
Source: BMC Evol Biol. 2020 Aug 18;20:104. doi: 10.1186/s12862-020-01664-x (PMC7433143; doi:10.1186/s12862-020-01664-x)
Supplement: Supplementary file 8 — Additional file 8: Tables S4. Listings of venom gland (a) and silk gland (b) specific microbiota. [file 12862_2020_1664_MOESM8_ESM.pdf]

Tables S4. Listings of venom gland (a) and silk gland (b) specific microbiota

Table 4a: Venom Gland Specific Microbiota

Taxa	Spider Species
Aerococcaceae	*L. geometricus*
Arenimonas	*L. hesperus*
Planctomycetes BD7-11	*L. mactans*
Pseudoclavibacter	*L. mactans*
Idiomarina	*L. mactans*
Acidimicrobiales	*L. mactans*
Myxococcales BIrii41	*S. grossa*
Cytophagaceae	*S. grossa*
Deltaproteobacteria NB1-j	*S. grossa*
Ramlibacter	*S. grossa*
Rhodocyclaceae	*S. grossa*
Pseudolabrys	*S. grossa*
Saprospiraceae	*S. grossa*
SAR324 clade (Marine group B) - uncultured delta proteobacterium	*S. grossa*
Tabrizicola	*S. grossa*
Nannocystis	*S. grossa*
Candidatus Moranbacteria	*S. grossa*
Clostridiaceae 1	*S. grossa*
Emticicia	*S. grossa*
Alphaproteobacteria Incertae Sedis	*S. grossa*
Clostridiisalibacter	*S. grossa*
Pseudonocardiaceae	*S. grossa*
Gemmatirosa	*S. grossa*
Edaphobacter	*P. tepidariorum*
Ruminococcaceae UCG-005	*P. tepidariorum*
Angustibacter	*P. tepidariorum*
Nostoc	*P. tepidariorum*
Frankiales	*P. tepidariorum*
Desulfosporosinus	*P. tepidariorum*
Truepera	*P. tepidariorum*
Asticcacaulis	*P. tepidariorum*
Paenalcaligenes	*P. tepidariorum*
Blautia	*P. tepidariorum*
Lachnoanaerobaculum	*P. tepidariorum*
Leadbetterella	*P. tepidariorum*
Longimicrobiaceae	*P. tepidariorum*
Desulfovermiculus	*P. tepidariorum*
Sphingobacteriales PHOS-HE51	*P. tepidariorum*
Elusimicrobia Lineage IV	*P. tepidariorum*
Rikenellaceae dgA-11 gut group	*P. tepidariorum*
Alloprevotella	*P. tepidariorum*
Curtobacterium	*P. tepidariorum*
Haliea	*P. tepidariorum*
Caulobacteraceae	*P. tepidariorum*
Pseudoxanthomonas	*P. tepidariorum*
Bilophila	*P. tepidariorum*
Cryobacterium	*P. tepidariorum*
Bacillaceae	*P. tepidariorum*
Solirubrobacterales 0319-6M6	*P. tepidariorum*
Variovorax	*L. mactans & S. grossa*
Psychrobacter	*L. geometricus & P. tepidariorum*

Table 4b: Silk Gland Specific Microbiota

Taxa	Spider Species
Rickettsiales SM2D12	*L. geometricus*
Carnobacteriaceae	*L. geometricus*
Lachnospiraceae NK3A20 group	*L. mactans*
Sphingobacteriales NS11-12 marine group	*S. grossa*
Tepidisphaeraceae	*S. grossa*
Devosia	*S. grossa*
Betaproteobacteria SC-I-84	*S. grossa*
Shewanella	*S. grossa*
Sporichthyaceae	*S. grossa*
Gaiellales	*S. grossa*
Myxococcales KD3-10	*S. grossa*
Rheinheimera	*S. grossa*
Myxococcales	*S. grossa*
Bacillaceae	*P. tepidariorum*
Mogibacterium	*P. tepidariorum*
Verrucomicrobia S-BQ2-57 soil group	*P. tepidariorum*
Abiotrophia	*P. tepidariorum*
Comamonadaceae	*P. tepidariorum*
Candidatus Peribacteria uncultured sludge bacterium	*P. tepidariorum*
Oligoflexus	*P. tepidariorum*
[Eubacterium] hallii group	*P. tepidariorum*
Intrasporangiaceae	*P. tepidariorum*
Acidimicrobiales	*P. tepidariorum*
Coprobacter	*P. tepidariorum*
Propionibacteriaceae	*P. tepidariorum*
Planococcaceae	*P. tepidariorum*
Acidobacteria Subgroup 6	*P. tepidariorum*
Filimonas	*P. tepidariorum*
Chloroflexi TK10	*P. tepidariorum*
Candidatus Peribacteria	*S. grossa & P. tepidariorum*
