# Supplementary material for: Spider phylosymbiosis: divergence of widow spider species and their tissues’ microbiomes
Source: BMC Evol Biol. 2020 Aug 18;20:104. doi: 10.1186/s12862-020-01664-x (PMC7433143; doi:10.1186/s12862-020-01664-x)
Supplement: Supplementary file 9 — Additional file 9: Table S5. Listings of ovary gland (a) and fat tissue (b) specific microbiota. [file 12862_2020_1664_MOESM9_ESM.pdf]

Table S5. Listings of ovary gland (a) and fat tissue (b) specific microbiota

Table 5a: Ovary Specific Microbiota	
**Taxa**	**Spider Species**
Spirochaeta 2	*L. geometricus*
Pseudarthrobacter	*L. geometricus*
Rhizobiales A0839	*L. geometricus*
Sphingobacteriales AKYH767	*L. geometricus*
Sideroxydans	*L. geometricus*
SR1 (Absconditabacteria)	*L. hesperus*
Lysobacter	*L. mactans*
Blastocatellaceae (Subgroup 4)	*L. mactans*
Roseomonas	*S. grossa*
Candidatus Yanofskybacteria bacterium GW2011 GWA2 41 22	*S. grossa*
Peredibacter	*S. grossa*
Vampirovibrionales	*S. grossa*
Myxococcales mle1-27	*S. grossa*
Candidatus Azambacteria	*S. grossa*
Woodsholea	*S. grossa*
Thermomicrobia JG30-KF-CM45	*P. tepidariorum*
Chloroflexi KD4-96	*P. tepidariorum*
Verrucomicrobia WCHB1-41	*P. tepidariorum*
Coxiella	*P. tepidariorum*
Beggiatoa	*P. tepidariorum*
Ignavibacteriales SR-FBR-L83	*P. tepidariorum*
Candidatus Falkowbacteria	*L. geometricus & L. mactans*

Table 5b: Fat Tissue Specific Microbiota	
**Taxa**	**Spider Species**
Ktedonobacteraceae	*L. geometricus*
Dyadobacter	*L. geometricus*
[Eubacterium] coprostanoligenes group	*L. geometricus*
Peptococcaceae	*L. geometricus*
Megamonas	*L. hesperus*
Fusobacteriales	*L. hesperus*
Tolumonas	*L. hesperus*
Gaiellales	*S. grossa*
Halanaerobiales ODP1230B8.23	*S. grossa*
Clostridium sensu stricto 12	*S. grossa*
Solibacillus	*S. grossa*
Oligoflexaceae	*S. grossa*
Solirubrobacterales FFCH11085	*S. grossa*
Ruminococcus 1	*S. grossa*
Candidatus Solibacter	*S. grossa*
Eubacteriaceae	*S. grossa*
Actinobacillus	*P. tepidariorum*
Pusillimonas	*P. tepidariorum*
Spongiibacteraceae BD1-7 clade	*P. tepidariorum*
Ardenticatenales	*P. tepidariorum*
Geodermatophilaceae	*P. tepidariorum*
Actinobacteria MB-A2-108	*P. tepidariorum*
Peptococcus	*P. tepidariorum*
Gemmatimonas	*L. geometricus & P. tepidariorum*
