# Supplementary material for: Spider phylosymbiosis: divergence of widow spider species and their tissues’ microbiomes
Source: BMC Evol Biol. 2020 Aug 18;20:104. doi: 10.1186/s12862-020-01664-x (PMC7433143; doi:10.1186/s12862-020-01664-x)
Supplement: Supplementary file 10 — Additional file 10: Figure S4. Phylosymbiosis between Widow Phylogeny and Microbiota Dendrogram Relationships. Congruency between widow related spider phylogeny and beta-diversity dendrograms was measured via Robinson-Foulds Cluster (R-F Cluster) metric, where a value of 0 = complete congruence and 2 = incomplete incongruence. Matching Cluster (MC) Metric also accounts for incongruency between closely related branches of rooted trees and a value of 0 also indicates complete congruence between compared trees. Pink asterisks are utilized to denote phylosymbiotic relationships between the widow phylogeny and microbial dendrogram. [file 12862_2020_1664_MOESM10_ESM.pdf]

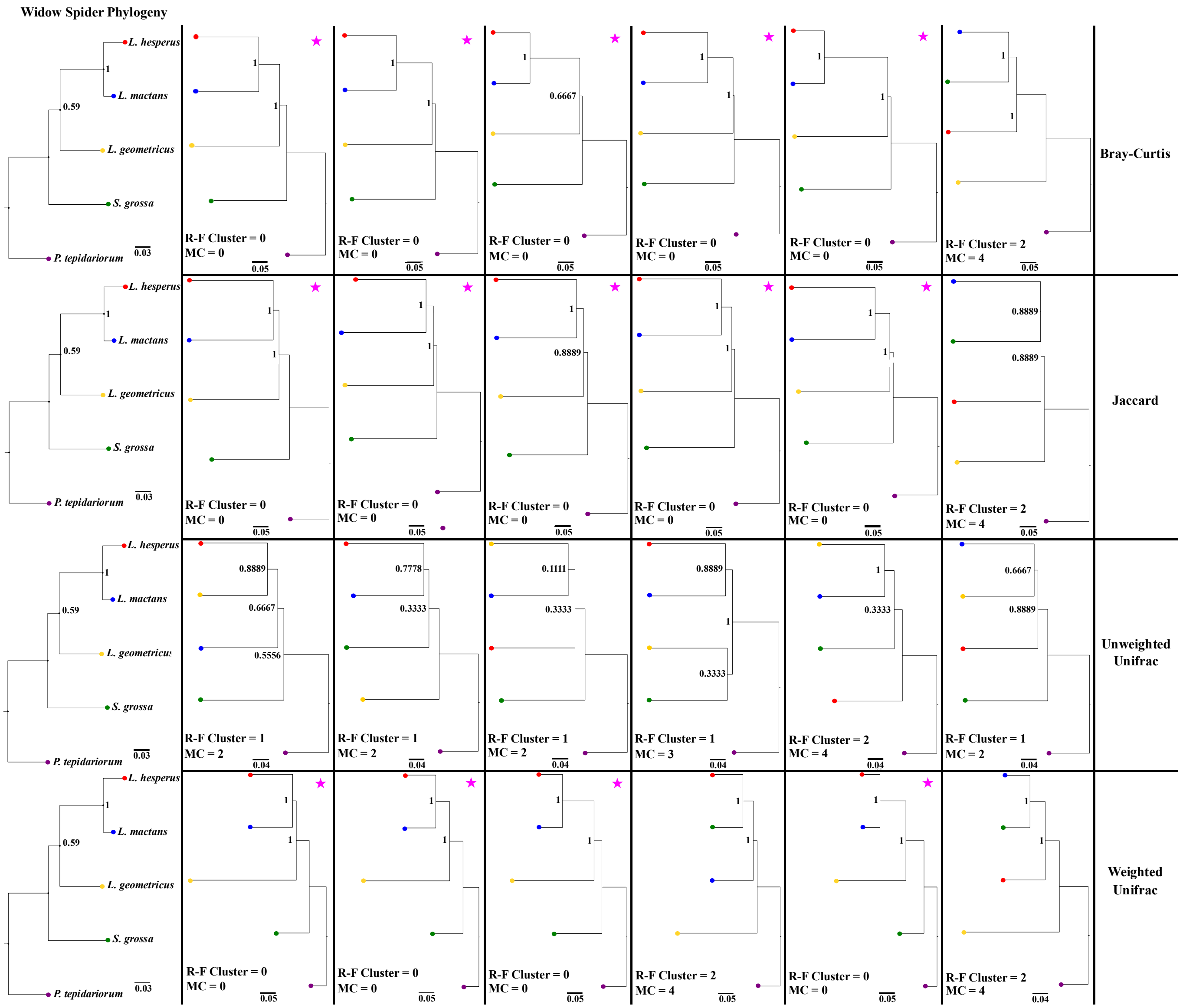

Figure S4. Phylosymbiosis between Widow Phylogeny and Microbiota Dendrogram Relationships. Congruency between widow related spider phylogeny and beta-diversity dendrograms was measured via Robinson-Foulds Cluster (R-F Cluster) metric, where a value of 0 = complete congruence and 2 = incomplete incongruence. Matching Cluster (MC) Metric also accounts for incongruency between closely related branches of rooted trees and a value of 0 also indicates complete congruence between compared trees. Pink asterisks are utilized to denote phylosymbiotic relationships between the widow phylogeny and microbial dendrogram.
