# Supplementary material for: Spider phylosymbiosis: divergence of widow spider species and their tissues’ microbiomes
Source: BMC Evol Biol. 2020 Aug 18;20:104. doi: 10.1186/s12862-020-01664-x (PMC7433143; doi:10.1186/s12862-020-01664-x)
Supplement: Supplementary file 11 — Additional file 11: Table S6. Microbial transcripts detected in RNA sequencing datasets from silk, venom and/ or ovary glands. [file 12862_2020_1664_MOESM11_ESM.pdf]

Table S6. Microbial transcripts detected in RNA sequencing datasets from silk, venom and/ or ovary glands.

RNA-Seq Data: Microbial Transcripts in Silk, Venom and/or Ovary Glands					
**Taxa Name**	**Taxa ID**	***P. tepidariorum***	***S. grossa***	***L. geometricus***	***L. hesperus***
Acidimicrobiaceae	84994	-	-	Silk Only	-
Acidipropionibacterium	1912215	-	Ovary Only	-	-
Acidovorax	12916	Silk & Venom	-	-	-
Actinobaculum	76833	-	Ovary Only	-	-
Actinomyces	1654	-	Venom & Ovary	-	-
Arthrospira	35823	-	Ovary Only	-	-
Alicycliphilus	201096	Venom Only	-	-	-
Atopobium	1380	-	-	-	Venom Only
Azospira	146937	Venom Only	-	-	-
Bordetella	517	Silk & Venom	-	-	-
Bradyrhizobiaceae	41294	-	-	-	Silk Only
Brochothrix	2755	-	-	-	Ovary Only
Campylobacter	194	-	-	Silk Only	-
Candidatus Carsonella	114185	-	-	-	Venom Only
Candidatus Riesia	401618	-	-	Venom Only	-
Capnocytophaga	1016		Ovary Only	-	Ovary Only
Cardiobacterium	2717	-	Venom Only	Silk Only	-
Chlamydia	810	-	Venom & Ovary	-	Venom Only
Christensenella	990721	-	-	-	Venom Only
Chryseobacterium	59732	Venom Only	-	-	-
Citrobacter	544	Venom Only	-	-	-
Comamonas	283	Silk & Venom	-	-	-
Commensalibacter	1079922		-	-	Ovary Only
Cutibacterium	1912216	-	Venom & Ovary	Venom Only	Venom & Ovary
Dechloromonas	73029	Silk & Venom	-	-	-
Dolosigranulum	29393	-	Venom & Ovary	-	Venom & Ovary
Elizabethkingia	308865	Silk & Venom	-	Venom Only	-
Empedobacter	59734	-	Venom Only	-	-
Enhydrobacter	212791	Venom Only	-	-	-
Enterobacter	547	Silk & Venom	-	-	-
Exiguobacterium	33986	-	-	Silk Only	-
Finegoldia	150022	-	Venom Only	-	-
Flavihumibacter	1004301	-	Silk Only	-	-
Fusobacterium	848	-	-	Silk Only	-
Geobacter	28231	Ovary Only	-	-	
Granulicatella	117563	-	-	Silk Only	-
Herbaspirillum	963	-	-	Venom Only	-
Kocuria	57493				
Lactobacillus	1578	-	Silk Only	-	-
Melaminivora	1649468	Venom Only	-	-	-
Mycobacterium	1763	-	-	-	Venom Only
Ochrobactrum	528	-	-	-	Venom Only
Neisseria	482	-	Ovary Only	-	-
Paenibacillus	44249	Venom Only	-	-	-
Pantoea	53335	Venom Only	-	-	-
Parvimonas	543311	-	-	Venom Only	-
Peptoniphilus	162289	Venom Only	Ovary Only	-	Ovary Only
Prevotella	838	-	Ovary Only		
Polynucleobacter	44013	Venom Only	-	-	-
Porphyromonas	836	-	-	Silk Only	-
Pseudoxanthomonas	83618	-	Venom Only	-	-
Rothia	32207	-	Venom & Ovary	Silk & Venom	Ovary Only
Salmonella	590	-	-	Ovary Only	Venom & Ovary
Serratia	613	-	Venom & Ovary	Venom & Ovary	Venom & Ovary
Sphingobacterium	28453	Venom Only	-	-	-
Tsukamurella	2060	Silk & Venom	-	-	-
Xanthomonas	338	Venom Only	-	-	-
Xenorhabdus	626	-	-	-	Venom Only
